# Semi-transparent graphite films growth on Ni and their double-sided polymer-free transfer

**DOI:** 10.1038/s41598-020-71435-7

**Published:** 2020-09-07

**Authors:** Geetanjali Deokar, Alessandro Genovese, Sandeep G. Surya, Chen Long, Khaled N. Salama, Pedro M. F. J. Costa

**Affiliations:** 1 Physical Science and Engineering Division, King Abdullah University of Science and Technology (KAUST), Thuwal, 23955‐6900 Saudi Arabia; 2 Core Labs, King Abdullah University of Science and Technology, Thuwal, 23955-6900 Saudi Arabia; 3 Sensors lab, Advanced Membranes and Porous Materials Center, Computer, Electrical and Mathematical Science and Engineering Division, King Abdullah University of Science and Technology, Thuwal, 23955-6900 Saudi Arabia

**Keywords:** Nanoscale materials, Synthesis and processing

## Abstract

Nanorange thickness graphite films (NGFs) are robust nanomaterials that can be produced via catalytic chemical vapour deposition but questions remain regarding their facile transfer and how surface topography may affect their application in next-generation devices. Here, we report the growth of NGFs (with an area of 55 cm^2^ and thickness of ~ 100 nm) on both sides of a polycrystalline Ni foil and their polymer-free transfer (front- and back-side, in areas up to 6 cm^2^). Due to the catalyst foil topography, the two carbon films differed in physical properties and other characteristics such as surface roughness. We demonstrate that the coarser back-side NGF is well-suited for NO_2_ sensing, whereas the smoother and more electrically conductive front-side NGF (2000 S/cm, sheet resistance − 50 Ω/sq) could be a viable conducting channel or counter electrode in solar cells (as it transmits 62% of visible light). Overall, the growth and transfer processes described could help realizing NGFs as an alternative carbon material for those technological applications where graphene and micrometer-thick graphite films are not an option.

## Introduction

Graphite is a widely used industrial material. It is remarkable that, with its relatively low mass density and properties such as high in-plane thermal and electrical conductivities, graphite is also very stable in thermally and chemically harsh environments^1,2^. In the form of flakes, graphite is a well-known source material for graphene research^3^. When processed as a film, it can be used in a broad range of applications, including heat sinks for electronic devices (e.g., smartphones)^4–7^, as an active material in sensors^8–10^, for electromagnetic interference shielding^11,12^ and extreme ultraviolet lithography pellicles^13,14^, conducting channels in solar cells^15,16^. For all these applications, it would be significantly advantageous if nanorange thickness graphite films (NGFs), with a controlled thickness of < 100 nm over a large area, could be produced and transferred easily.

Graphite films have been produced using varied approaches. In one case, intercalation and expansion, followed by exfoliation, to produce graphene flakes have been used^10,11,17^. Further processing of the flakes to films of desired thickness is needed, often adding up to several days to complete compact sheets of graphite. One other process is to start from graphitizable solid precursors. In industry, polymer sheets undergo carbonization (at 1,000–1,500 °C) and are subsequently graphitised (at 2,800–3,200 °C), resulting in a well-structured layered material. While the quality of these films is high, the energy consumption is considerable^1,18,19^ and the minimal thickness is limited to a few micrometers^1,18–20^.

Catalytic chemical vapour deposition (CVD) is a known method to produce graphene and ultrathin graphite films (< 10 nm) with high structural quality and at reasonable costs^21–27^. However, in comparison with the graphene and ultrathin-graphite film growth^28^, the large-area growth and/or applications of NGF using CVD remain less explored^11,13,29–33^.

CVD-grown graphene and graphite thin films often require transferring onto a functional substrate^34^. These films transfer includes two main approaches^35^: (1) etch-free transfer^36,37^, (2) etching-based (support assisted) wet-chemical transfer^14,34,38^. Each approach has some advantages and disadvantages, and they need to be selected based on the target application, as discussed elsewhere^35,39^. For the graphene/graphite thin films grown on catalytic substrates, the transfer via a wet-chemical process (where poly(methyl methacrylate) (PMMA) is the most popular support layer used) remains the preferred option^13,30,34,38,40–42^. Yoo et al. mentioned no use of polymer for the transfer of NGFs (sample size ~ 4 cm^2^)^25,43^; however, no details on the sample stability and/or handling during the transfer were provided. The polymer assisted wet-chemical process consists of several stages, including the application and subsequent removal of the sacrificial polymer layer^30,38,40–42^. There are drawbacks to this procedure, for instance, polymer residues may alter the as-grown film properties^38^. Additional processing can remove polymer residues, but these added steps increase the cost and time of film manufacturing^38,40^. During the CVD growth, the graphene layers are deposited not only on the front-side of the catalyst foil (the one facing the vapour stream) but also on its back-side. Yet, the latter is considered waste material and promptly removed by a mild plasma treatment^38,41^. Retrieving this film could be of interest to maximise production output, even if its quality is lower than that of the front-side carbon film.

Here, we report on the preparation of double-sided, wafer-scale, high structural quality NGF growth on polycrystalline Ni foils via CVD method. An evaluation is made on how the front- and back-sides surface roughnesses of the foil affect the NGF morphology and structure. We also demonstrate the polymer-free economic and environmentally friendly transfer of the NGFs, from both sides of the Ni foil on versatile substrates, and indicate how the front- and back-side films are suitable for different applications.

## Results and discussion

In the following sections, varied graphite film thicknesses are discussed on the basis of the number of stacked graphene layers: (i) single-layer graphene (SLG, 1 layer), (ii) few-layer graphene (FLG, < 10 layers), (iii) multi-layer graphene (MLG, 10–30 layers), and (iv) NGF (~ 300 layers). The last was the most common thickness observed in terms of area percentage (~ 97% areas in 100 µm^2^)^30^. Therefore, the entire film is simply referred to as NGF.

### Growth of FS- and BS-NGF

**Figure 1:**
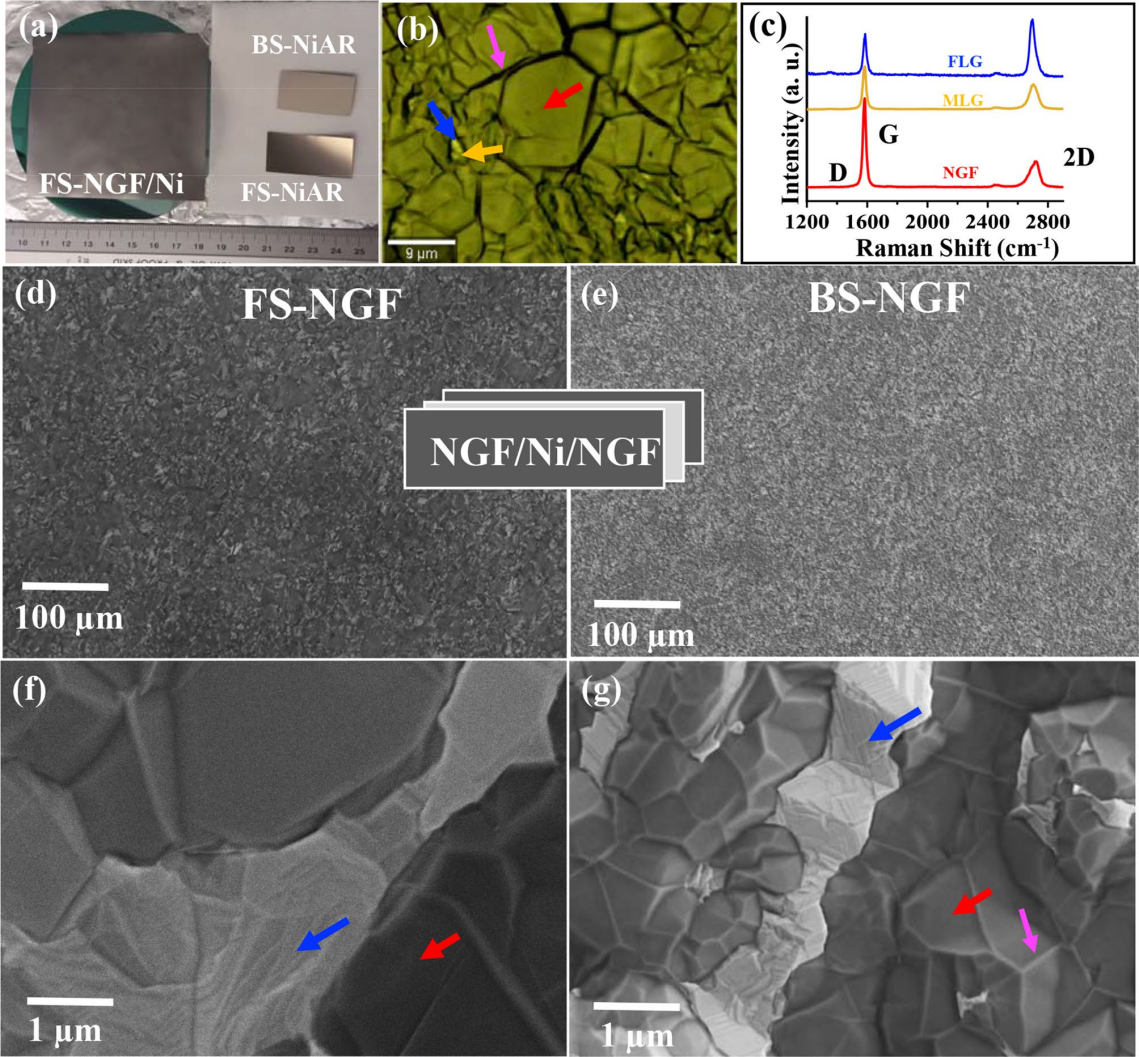
Comparison of FS- and BS-NGF for NiAG: (**a**) Typical NGF sample (NiAG) photo showing wafer-scale NGF growth (55 cm^2^) along-with samples of as-received BS- and FS-Ni foils, (**b**) Optical microscopy image of FS-NGF/Ni, (**c**) Typical Raman spectra recorded on different positions in panel-**b**, (**d**,**f**) SEM images with different magnification on FS-NGF/Ni, (**e**,**g**) SEM images with different magnification BS-NGF/Ni. Blue arrow indicate FLG areas, orange arrow indicate MLG areas (in vicinity to FLG areas), red arrow indicate NGF areas, and magenta arrow indicate fold.

Polycrystalline Ni foils used for graphene and graphite film synthesis have different textures due to their manufacturing and subsequent processing. We recently reported on the NGF growth process optimisation studies^30^. We showed that the processing parameters, such as annealing times and chamber pressures during the growth step have crucial roles in obtaining NGF with uniform thickness. Here, we further explored the NGFs growth for the polished front-side (FS) and unpolished back-side (BS) Ni foil surfaces (Fig. 1a). Three types of FS and BS samples were studied, as listed in Table 1. Uniform NGF growth on both sides of the Ni foil (NiAG) was noted by eye inspection as the host Ni substrate changed colour from its characteristic metallic silver-grey to dull grey (Fig. 1a); this was confirmed via Raman microscopy measurements at various positions on sample (Fig. 1b,c). Typical Raman spectra for FS-NGF, observed in bright areas and marked in Fig. 1b by red, blue, and orange arrows, are displayed in Fig. 1c. Characteristic graphitic Raman signature peaks, G (1683 cm^−1^) and 2D (2,696 cm^−1^) confirmed the growth of highly crystalline NGF (Fig. 1c, Table SI1). In the overall film, the dominant presence of Raman spectra with intensity ratio (I_2D_/I_G_) ~ 0.3, along with the rare appearance of Raman spectra with I_2D_/I_G_ = 0.8, was observed. The absence of a defect related peak (D = 1,350 cm^−1^) in the overall film indicated a high-quality NGF growth. Similar Raman results were obtained on the BS-NGF samples (Figure SI1 a and b, Table SI1).

**Table 1:** A list of samples used in this study and their short names based on the treatment done.

Processing Ni foil (25 µm)	Pre- annealing time (A_t_ , min)	Chamber pressure during growth (P_c_ , mbar)	Growth time (G_t_ , min)	Short name
None, As-received	–	–	–	**NiAR**
Annealing	5	–	–	**NiA**
Annealing and NGF Growth	5	500	5	**NiAG**

Other process parameters such as annealing and growth temperature (900 °C), chamber pressure during annealing (10 mbar), gas flow ratio were kept constant. We have characterized both FS and BS for each types of sample.

Achieving reasonable NGF thickness control over a large area remains a challenging task, due to the growth dependence on host substrate thickness, crystal size, orientation, and grain boundaries^20,34,44^. In this study, uniform NGF (down to a mm^2^ scale) was observed by scanning electron microscopy (SEM) imaging, on both the FS- and BS-Ni foils (Fig. 1d,e, respectively), using similar optimized growth conditions as those previously reported by us^30^. This process results in 0.1 to 3% bright areas in 100 µm^2^^30^. In the following sections, we have presented the results for both types of regions. High-magnification SEM images showed a few bright contrast regions present on both sides (Fig. 1f,g), indicating to the presence of FLG and MLG regions^30,45^. This was also confirmed by Raman (Fig. 1c) and TEM results (discussed later in “FS-NGF: structure and properties” section). FLG and MLG areas, observed on both FS- and BS-NGF/Ni (front- and back-side NGF grown on Ni) samples, might have grown on large Ni(111) grains formed during preannealing^22,30,45^. The presence of folds (Fig. 1b, marked by a magenta arrow) was observed on both sides. These folds are commonly observed for CVD-grown graphene and graphite films due to a large thermal expansion coefficient difference between graphite and the Ni substrate^30,38^.

FS-NGF samples were flatter than BS-NGF samples (Figures SI1), as confirmed by AFM imaging (Figure SI2). The root mean square (RMS) roughness values for FS-NGF/Ni (Figure SI2c) and BS-NGF/Ni (Figure SI2d) were 82 and 200 nm (measured over 20 × 20 µm^2^), respectively. The higher roughness could be understood based on the surface analysis of the as-received Ni (NiAR) foil (Figure SI3). SEM images of FS- and BS-NiAR, displayed in Figures SI3a–d, indicate different surface topographies with the polished FS-Ni foil possessing spherical nano- and micron-size granules, whereas the unpolished BS-Ni foil shows stepped grains with highs and dips. The low- and high-resolution images of the annealed Ni foil (NiA) are displayed in Figures SI3e–h. In these figures, we can observe the presence of Ni grains of a few microns sized on both side surfaces of the Ni foil (Figures SI3e–h). The large grains could be of Ni(111) surface orientation, as reported previously^30,46^. There was a significant difference in terms of Ni foil topography between FS- and BS-NiA. The higher roughness of BS-NGF/Ni was associated with the unpolished BS-NiAR surface, which remained significantly rough even after annealing (Figure SI3). This type of surface characterization performed prior to the growth process, provides a means of controlling the roughness of the graphene and graphite film. It should be noted that the host substrate undergoes some grain restructuring during graphene growth, which reduces the grain size slightly and increases the substrate surface roughness to some extent when compared to annealed catalyst foils and films^22^.

Fine-tuning the substrate’s surface roughness, annealing time (grain size)^30,47^ and precipitation control^43^ would help in reducing the areal NGF thickness uniformity down to µm^2^ and/or even nm^2^ scale (i.e., with thickness variations of a few nanometers). To control substrate surface roughness, techniques such as electropolishing of the as received Ni foils could be considered^48^. Then the pre-treated Ni foils could be subjected to lower annealing temperature (< 900 °C)^46^ and time (< 5 min) to avoid large Ni(111) grain formation (which favors FLG growth).

### Polymer-free transfer of FS- and BS-NGF

**Figure 2:**
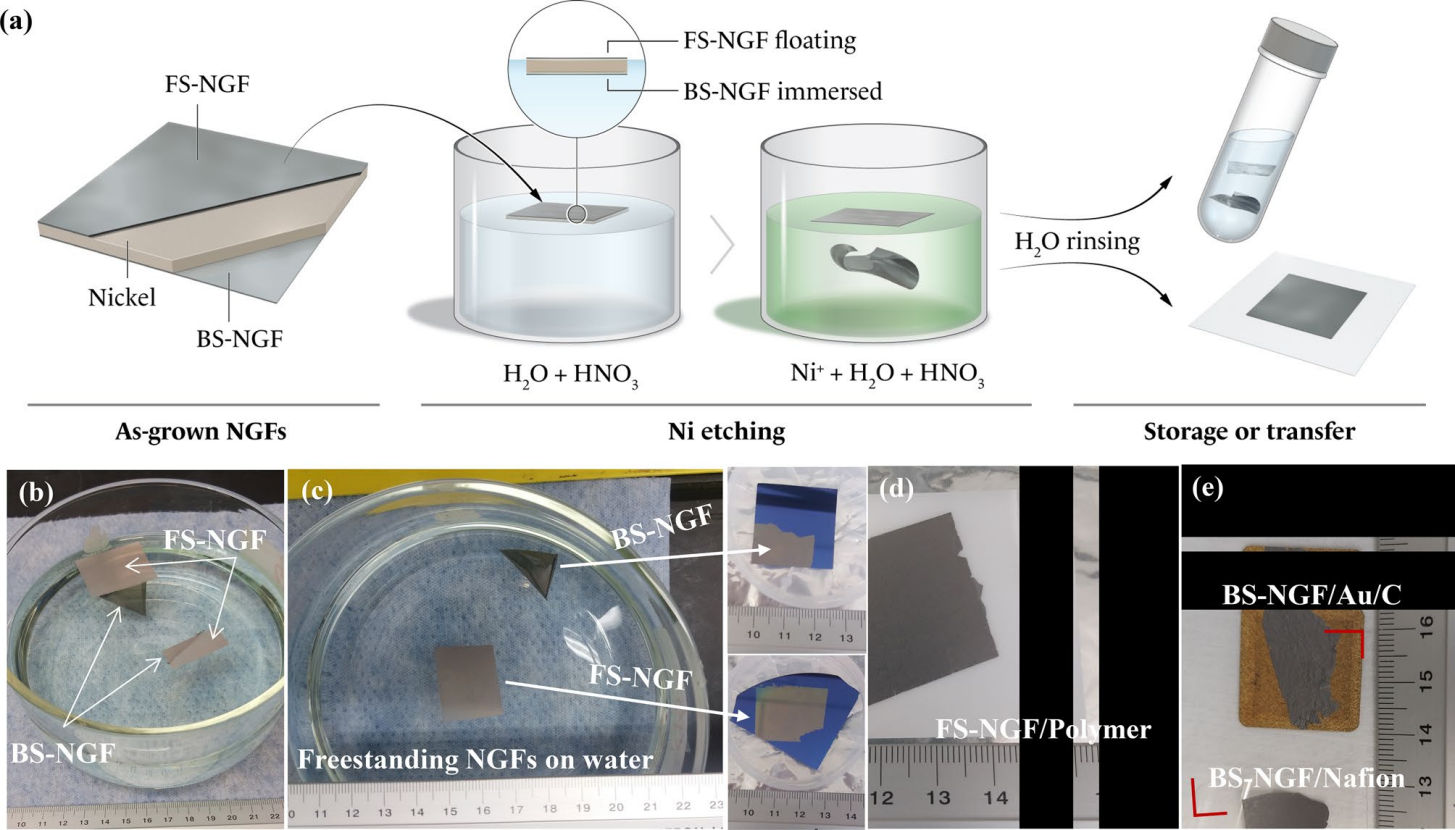
Polymer-free wet chemical transfer process for NGFs grown on Ni foil: (**a**) schematic presentation of the process (see Figure SI4 for more details), (**b**) digital photo of NGFs detached after Ni etching (2 samples), (**c**) example of FS- and BS-NGF transfer on SiO_2_/Si substrate, (**d**) FS-NGF transfer on opaque polymer substrate, (**e**) BS-NGF (broken into two pieces) from same sample as that of panel-**d**, transferred on Au coated C-paper and Nafion (flexible and transparent substrate, edges marked by red angles).

SLG and FLG graphene cannot withstand acid and water surface tensions, requiring a mechanical support layer during the wet-chemical transfer process^22,34,38^. Unlike the polymer-supported wet chemical transfer of monolayer graphene^38^, we found that both sides of the as-grown NGFs could be transferred without polymer support, as schematically represented in Fig. 2a (for more details refer to Figure SI4a). Transferring of the NGF to a given substrate was initiated by wet-etching the underlying Ni film^30,49^. As-grown NGF/Ni/NGF samples were kept floating overnight in 15 ml of 70% HNO_3_ diluted in 600 ml of deionised (DI) water. After complete dissolution of the Ni foil, the FS-NGF remained flat and floating on the liquid surface, as did the NGF/Ni/NGF sample, whearas the BS-NGF had become immersed (Fig. 2a,b). The detached NGFs were then carefully rinsed by transferring them from one beaker of fresh DI water to another, an action repeated four to six times via a concave glass dish. Finally, both FS- and BS-NGFs were placed on the desired substrate (Fig. 2c).

Note that SLG transfer done with a wet-chemical transfer technique needs a total process time of 20–24 h^38^. With the polymer-free transfer technique demonstrated here (Figure SI4a), the total process time for NGFs transfer is significantly less (~ 15 h). This process includes: (step-1) preparation of the etchant solution and floating the sample on it (~ 10 min), then wait overnight for Ni etching (~ 7,200 min), (step-2) DI water rinsing, (step-3) store in DI water or transfer on to the target substrate (20 min). Water trapped between the NGF and bulk substrate was removed by capillary action (with the use of a blotting paper)^38^, followed by a natural-dry process to remove the remaining water drops (~ 30 min) and lastly the sample was baked for 10 min in a vacuum furnace (10^–1^ mbar) at 50–90 °C (60 min)^38^.

It is known that graphite can withstand the presence of water or air at fairly high-temperatures (≥ 200 °C)^50–52^. We tested the samples after storing them for several days to one year, in DI water at room temperature and in sealed bottles (Figure SI4) by Raman spectroscopy, SEM, and XRD. There was no noticeable degradation. In Fig. 2c, freestanding FS-NGF and BS-NGF in DI water are shown. We captured them on a SiO_2_(300 nm)/Si substrate, as shown in the outsets of Fig. 2c. Additionally, as shown in Fig. 2d,e, the continuous NGFs could be transferred to various substrates, such as polymers (Thermalbright Polyamide from Nexolve and Nafion) and Au-coated carbon paper. The floating FS-NGF was placed on the target substrates rather easily (Fig. 2c, d). However, when wholly immersed in water, BS-NGF samples with areas greater than 3 cm^2^ were challenging to transfer. Generally, as they started rolling up in the water and sometimes broke into two or three pieces due to rough handling (Fig. 2e). Overall, we were able to achieve a polymer-free transfer of FS- and BS-NGF for samples with areas up to 6 and 3 cm^2^ (continuous one-piece transfer without cracks or structural damage from 6 cm^2^ as-grown NGF/Ni/NGF), respectively. Any remaining big or tiny pieces can be (easily visible in the etchant solution or DI water) fished on the desired substrate (~ 1 mm^2^, Figure SI4b, see sample transferred on Cu grid, as discussed in “FS-NGF: structure and properties” section) or stored for future use (Figure SI4). With this criterion, we estimate one can recover the NGFs with a yield up to ~ 98–99% (as-grown to after transfer).

**Figure 3:**
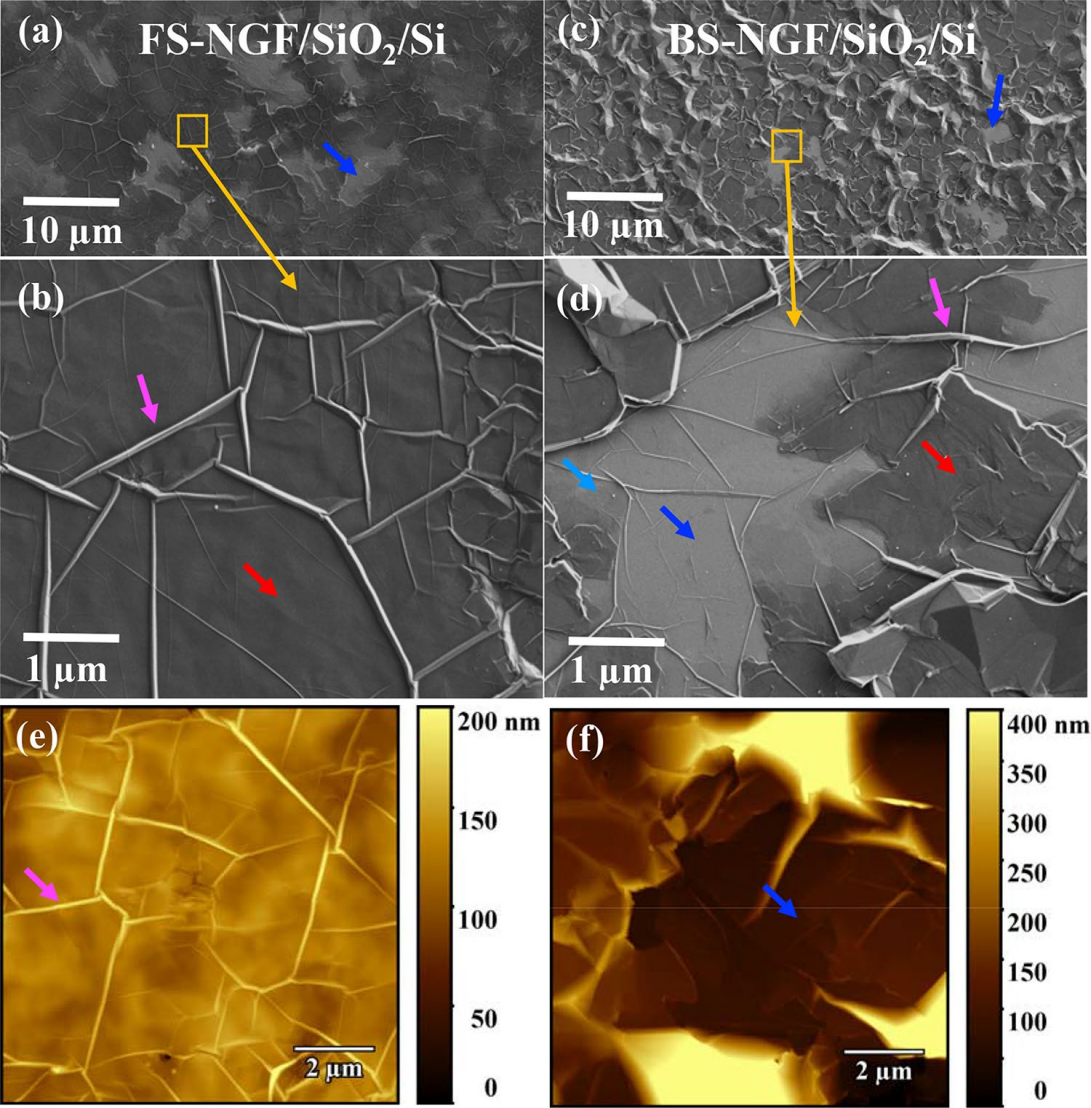
Polymer-free transferred NGF (NiAG) on SiO_2_/Si wafers (see Fig. **2****c**): (**a**,**b**) SEM images of transferred FS-NGF: low- and high-magnification (corresponding to typical area shown by orange square in panel-**a**). (**c**,**d**) SEM images of transferred BS-NGF: low- and high-magnification (corresponding to typical area shown by orange square in panel-**c**). (**e**,**f**) AFM images of transferred FS- and BS-NGFs. Blue arrow indicate FLG areas—bright contrast, Cyan arrow—blackish contrast MLG, red arrow—black contrast indicate NGF areas, and magenta arrow indicate fold.

The polymer-free transferred samples were analysed in detail. The surface morphology characterisation, obtained via optical microscopy (OM) and SEM imaging (Figure SI5 and Fig. 3) on the FS- and BS-NGF/SiO_2_/Si (Fig. 2c), revealed that these samples had been transferred without microscopically visible structural damage, such as cracks, holes, or rolled-up areas. The folds (Fig. 3b,d, marked by magenta arrows) on the as-grown NGF remained intact after the transfer process. Both FS- and BS-NGFs consisted of FLG regions (bright areas, marked by blue arrows in Fig. 3). Surprisingly, unlike the several broken areas commonly seen in the case of the polymer-assisted transfer of ultrathin graphite films^34^, the few micron-sized FLG and MLG areas (marked by cyan arrows in Fig. 3d) bridged to NGF were transferred without cracks and tears (Fig. 3). The mechanical integrity was further confirmed by TEM and SEM imaging of the NGF transferred to a Cu grid with lacey carbon, as discussed later (“FS-NGF: structure and properties”). The transferred BS-NGF/SiO_2_/Si was rougher than the FS-NGF/SiO_2_/Si with RMS values of 140 nm and 17 nm, respectively, as seen in Figures SI6a and b (20 × 20 µm^2^). The RMS value for the NGFs transferred onto a SiO_2_/Si substrate (RMS < 2 nm) was much lower (~ 3 times) than that of the the as-grown NGFs on Ni (Figure SI2), indicating the additional roughness could correspond to the Ni surface. Moreover, AFM imaging performed at the edge of the FS- and BS-NGF/SiO_2_/Si samples showed NGF thicknesses of 100 and 80 nm, respectively (Figure SI7). The lower thickness of BS-NGF could be a consequence of the surface not being exposed directly to precursor gases.

**Figure 4:**
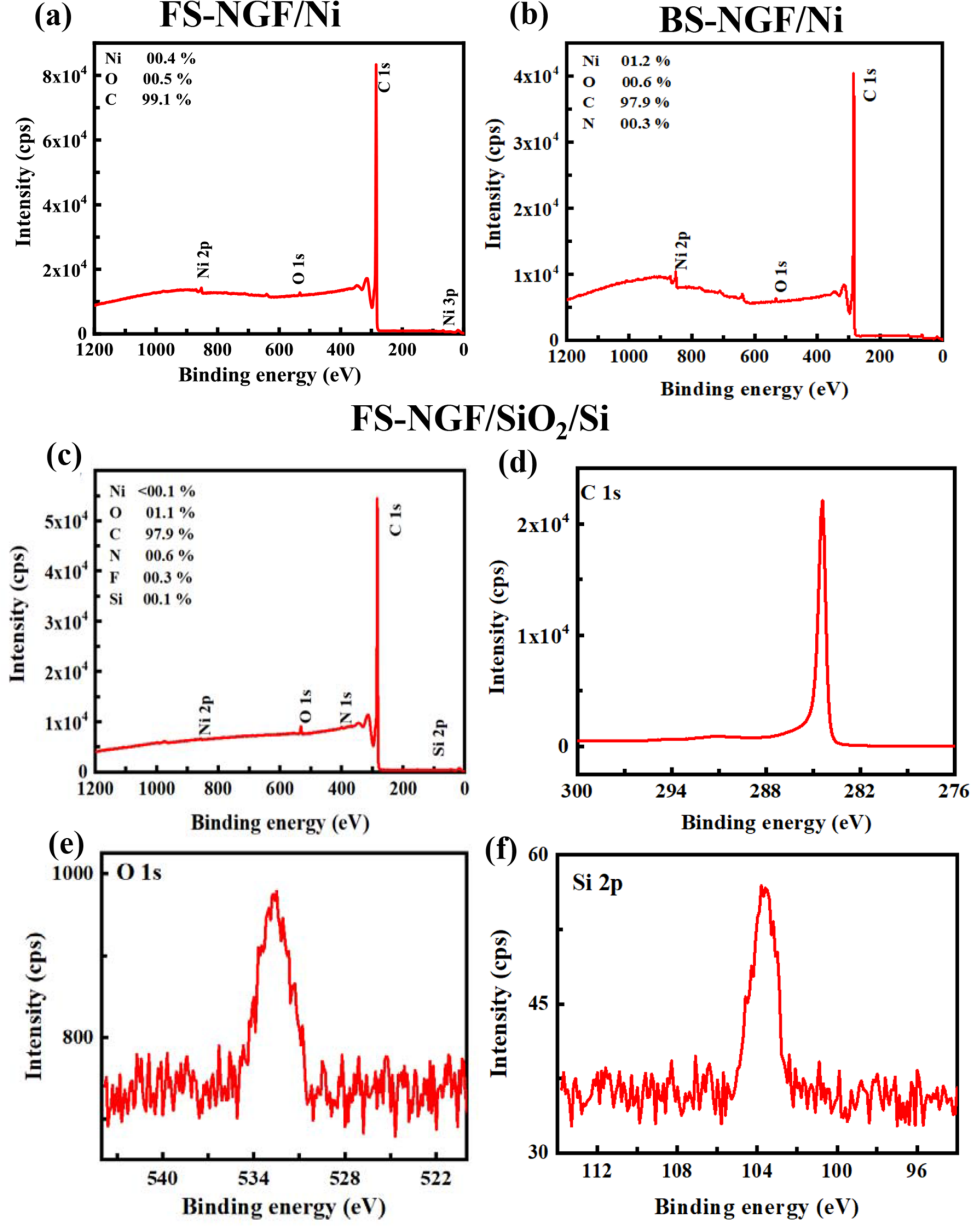
XPS results of NiAG: (**a**–**c**) Survey spectra with atomic composition of different elements for as-grown FS-NGF/Ni, BS-NGF/Ni and transferred FS-NGF/SiO_2_/Si, respectively. (**d**–**f** ) High-resolution spectra of the C 1 s, O1s, Si 2p core levels, respectively, for FS-NGF/SiO_2_/Si sample.

The chemical composition of the as-grown and transferred FS- and BS-NGFs was analysed by X-ray photoelectron spectroscopy (XPS) (Fig. 4). A weak peak was observed in the survey spectra (Fig. 4a,b) corresponding to the Ni substrate (850 eV) for the as-grown FS- and BS-NGF (NiAG). The peak was absent in the survey spectra of the transferred FS- and BS-NGF/SiO_2_/Si (Fig. 4c; similar results for BS-NGF/SiO_2_/Si are not shown), indicating no Ni contamination remained after transfer. In Fig. 4d–f, high-resolution spectra of the C 1 s, O 1 s, and Si 2p levels are shown for FS-NGF/SiO_2_/Si. The C 1 s binding energy for graphite is 284.4 eV^53,54^. The line-shape of the graphitic peak is generally regarded as asymmetrical, as observed in Fig. 4d^54^. The high-resolution C 1 s core-level spectrum (Fig. 4d) also confirmed a clean transfer (i.e., without polymer residues) in agreement with previous research^38^. The linewidths of the C 1 s spectra were 0.55 and 0.62 eV for the as-grown sample (NiAG) and after the transfer, respectively. These values were higher than that of SLG (0.49 eV for SLG on SiO_2_ substrates)^38^. However, these values were smaller than previously reported linewidths for highly-oriented pyrolytic graphene samples (~ 0.75 eV)^53–55^, indicating the absence of defective carbon sites in the current material. There were also no shoulder peaks in the C 1 s and O 1 s core-level spectra, excluding the need for performing deconvolution on high-resolution peaks^54^. There were π → π* satellite peak at approximately 291.1 eV, which is often observed in graphite samples^54^. Signals at 103 eV and 532.5 eV in the Si 2p and O 1 s core-level spectra, respectively, (see Fig. 4e,f) were ascribed to the SiO_2_ substrate^56^. XPS is a surface-sensitive technique, so the detected signals corresponding to Ni and SiO_2_, before and after NGF transfer, respectively, were assumed to originate from the FLG regions. Similar results were observed for the transferred BS-NGF sample (not shown).

The overall crystalline quality of the transferred NGFs was assessed using X-ray diffraction (XRD). Typical XRD diffraction pattern for the transferred FS- and BS-NGF/SiO_2_/Si (Figure SI8) showed the presence of the (0 0 0 2) and (0 0 0 4) diffraction peaks at 26.6° and 54.7°, similar to graphite. That confirmed the high crystalline quality of the NGF and corresponded to an interlayer spacing of d = 0.335 nm, retained after the transfer steps. The intensity of the (0 0 0 2) diffraction peak was ~ 30 times larger than that of (0 0 0 4), indicating the NGF crystal planes were well-aligned with the sample's surface.

Based on the SEM, Raman spectroscopy, XPS, and XRD results, the BS-NGF/Ni was found to be of equally high-quality as the FS-NGF/Ni, though it had a slightly higher RMS roughness (Figures SI2, SI5, and SI7).

A SLG with a polymer support layer as thin as 200 nm can float on the water surface. This setup is routinely used in the polymer assisted wet-chemical transfer processes^22, 38^. Graphene and graphite are hydrophobic (with 80–90° of contact angle)^57^. It has been reported that for both graphene and FLG, the potential energy surfaces are rather flat with low barriers (∼1 kJ/mol) in respect to the lateral motion of the water over the surface^58^. However, the calculated interaction energy of water with graphene and three graphene layers is about − 13 and − 15 kJ/mol, respectively^58^, pointing to a relatively weaker interaction of water with NGF (~ 300 layers) when compared to graphene. This could be one of the reasons why the freestanding NGF remains flat on the water surface while the freestanding graphene (floating on the water surface) curls-up and gets destructed. When the NGF is fully immersed in the water (same outcome for the rough and flat NGF), its edges get folded (Figure SI4). Expectedly, with full immersion, the NGF-water interaction energy is almost double (as compared to the floating NGF), and to maintain the contact angle high (hydrophobic nature), the NGF edges fold. We believe that strategies can be build up to avoid the curling of immersed NGF edges. One approach would be to use a mix of solvents, thereby regulating the wetting response of the graphitic films^59^.

The transfer of SLG onto various types of substrates via wet chemical transfer process has been reported previously. It is consensually accepted that there is a weak van der Waals force present between the graphene/graphite film and the substrate (whether rigid substrates such as SiO_2_/Si^38,41,46,60^, SiC^38^, Au^42^, Si pillars^22^ and, Cu grid with lacey carbon film^30,34^, or flexible substrates such as polyimide^37^). Here, we assume that the same type of interaction prevails. Upon mechanical handling (during characterization or storage in a vacuum and/or atmospheric conditions), we did not observe any damage or peeling off of the NGF for any of the substrates presented here (e. g., Fig. 2_,_ SI7, and SI9). Additionally, we did not observe SiC peaks in the XPS C 1 s core-level spectrum of the NGF/SiO_2_/Si samples (Fig. 4). These results indicating that there is no chemical bonding between the NGF and the target substrates.

### Properties and applications of FS- and BS-NGF

In the previous “Polymer-free transfer of FS- and BS-NGF” section, we demonstrated that the NGF could be grown and transferred from both sides of the Ni foil. These FS- and BS-NGFs were unequal regarding surface roughness, prompting us to explore the most appropriate applications for each type.

#### FS-NGF: structure and properties

**Figure 5:**
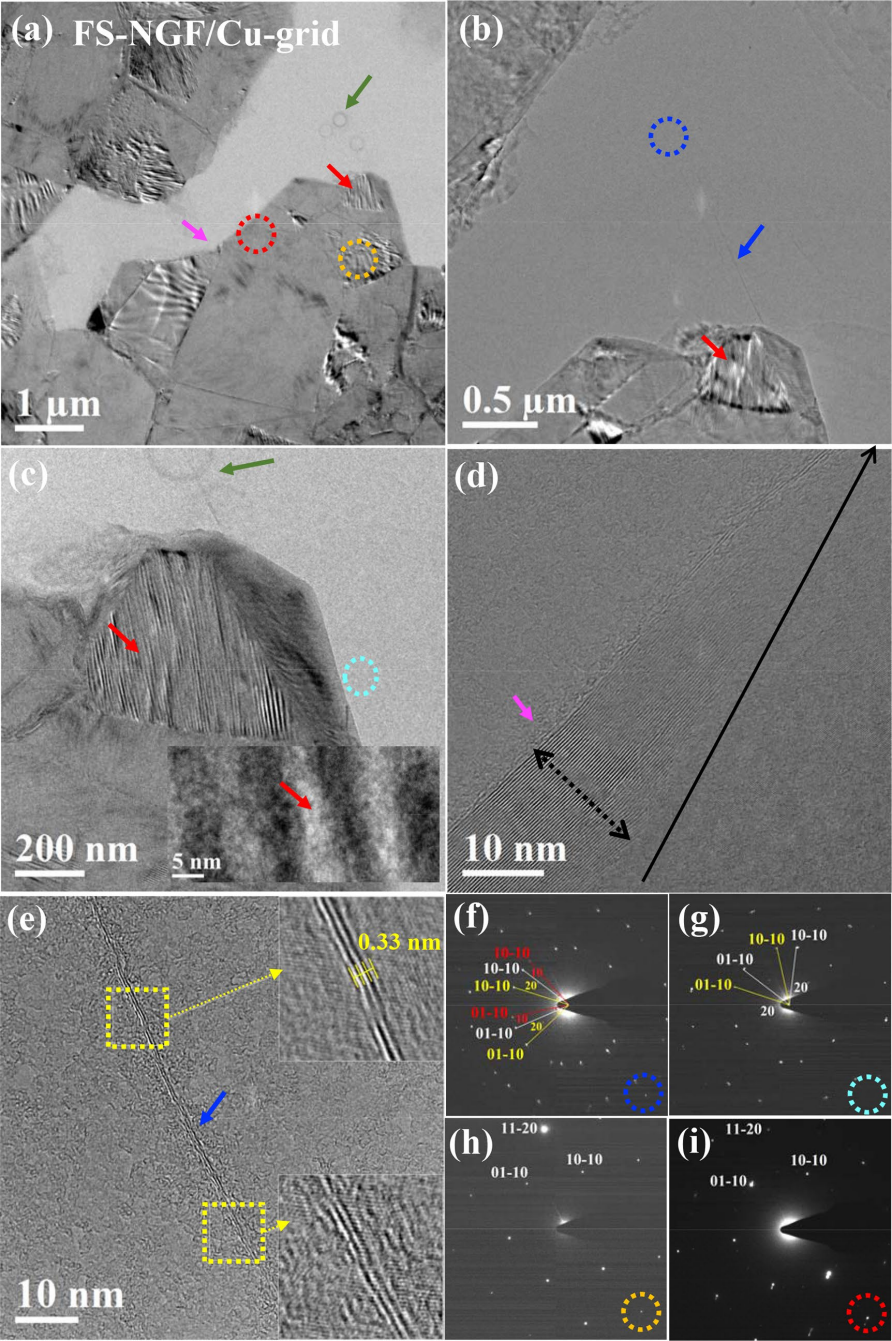
Plan-view TEM imaging of the polymer-free transferred NiAG sample on Cu grid with lacey carbon: (**a**,**b**) Low-magnification TEM images including NGF and FLG areas, (**c**–**e**) high-magnification images at different areas in panel-**a** and panel-**b** as marked by same color arrows. Green arrow in panel-**a** and **c** indicate circular damaged areas during beam-alignment. (**f**–**i**) SAED patterns at different areas marked by blue, cyan, orange, and red circles, respectively, in panel-**a** to panel-**c**.

Given the transparency and smoother surface of the FS-NGF, we investigated its local structure in greater detail, as well as its optical and electrical properties. The textural and structural characterisation of the polymer-free transferred FS-NGF were performed via transmission electron microscopy (TEM) imaging and selected area electron diffraction (SAED) pattern analysis. The corresponding results are shown in Fig. 5. Plan-view TEM imaging at a low magnification revealed the presence of NGF and FLG regions characterised by different electron contrast, namely darker and brighter regions, respectively (Fig. 5a). The film showed an overall good mechanical integrity and stability between the different NGF and FLG regions, which were well-bridged without damage or laceration, as also confirmed by SEM imaging (Fig. 3) and TEM investigation at higher magnification (Fig. 5c–e). In particular, Fig. 5d displays a bridging structure in the largest portion (position marked by a dotted black arrow in Fig. 5d) characterised by a triangle-like shape and consisting of a width of about 51 graphene layers with an interplanar distance of 0.33 ± 0.01 nm, which reduced further to a few graphene layers at the narrowest area (end of the solid black arrow in Fig. 5d).

Strip-like structures in Fig. 5c showing (marked by the red arrow) the vertical orientation of the graphitic lattice planes might be related to the formation of nanofolds (inset of Fig. 5c) alongside the film due to an excess of uncompensated tangential stress^30,61,62^. Under high-resolution TEM, these nanofolds^30^ revealed a crystalline orientation that differed from the rest of the NGF area; graphitic lattice basal planes were almost vertically oriented instead of horizontally, as in the rest of the thin film (inset of Fig. 5c). Similarly, the FLG regions occasionally showed the presence of folds as linear and narrow strips (marked by blue arrows) that appeared under low- and intermediate-magnification in Fig. 5b,e, respectively. The insets of Fig. 5e confirm the presence of two and three graphene layers (an interplanar distance of 0.33 ± 0.01 nm) in the FLG sector, which agrees well with our previous results^30^. Moreover, the recorded SEM images of polymer-free NGF transferred onto a Cu grid with lacey carbon film (after performing plan-view TEM measurements) are displayed in Figure SI9. The well-suspended FLG areas (marked by a blue arrow) along with a broken region in Figure SI9f. marked by a cyan arrow (at the edge of the transferred NGF) are intentionally presented, to demonstrate that the FLG regions can withstand the polymer-free transfer process. Thus, these images confirm that the mechanical integrity of the partially-suspended NGF (including FLG areas) was maintained even after rigorous handling and exposure to high vacuum environments during the TEM and SEM measurements (Figure SI9).

Thanks to the excellent flatness of the NGF (see Fig. 5a), it was not difficult to orient the flakes along their [0001] zone axis for SAED pattern analysis. Several regions (12 spots) of interest were identified for electron diffraction investigation, selected according to the local thickness of the film and their positions. In Fig. 5a–c, four of these typical regions are shown and marked with coloured circles (blue, cyan, orange, and red colour-coding). The SAED patterns of Figs. 5f and g were acquired from the FLG areas shown in Figs. 5b and c, respectively. These exhibited a hexagonal pattern similar to twisted graphene^63^. Specifically, Fig. 5f shows three superimposed patterns with identical [0001] zone axis orientation that were rotated by 10° and 20°, as highlighted by the angular mismatch of the three pairs of (10–10) reflections. Similarly, Fig. 5g shows two superimposed hexagonal patterns rotated by 20°. The two or three sets of hexagonal patterns in the FLG areas could have originated from the three in-plane or out-of-plane graphene layers rotated with respect to each other^33^. By contrast, the electron diffraction patterns of Fig. 5h,i, which correspond to the NGF regions shown in Fig. 5a, exhibited single [0001] patterns with an overall higher diffraction intensity of spots, consistent with larger material thickness. These SAED patterns are consistent with a thicker graphitic structure than FLG and an average common orientation, as deduced from the indexing^64^. The characterisation of crystal properties of NGFs indicated two or three superimposed graphite (or graphene) crystallites coexisted. Especially noticeable in the FLG regions, the crystallites had a degree of in-plane or out-of-plane misorientation. Graphite grains/layers with in-plane rotation angles of 17°, 22° and 25° were reported previously for NGF grown on Ni film^64^. The rotation angle values observed by this study are consistent with rotation angles (± 1°) observed previously for twisted BLG graphene^63^.

The electrical properties of the NGF/SiO_2_/Si were measured at 300 K over a 10 × 3 mm^2^ area. The electron carrier concentration, mobility, and electrical conductivity values were 1.6 × 10^20^ cm^−3^, 220 cm^2^ V^−1^ S^−1^ and 2000 S cm^−1^, respectively. The mobility and conductivity values for our NGF were similar to natural graphite^2^ and higher than commercially available highly-oriented pyrolytic graphite (produced at 3000 °C)^29^. The electron carrier concentration value observed was two orders of magnitude higher than the carrier concentration value (7.25 × 10^18^ cm^−3^) of recently reported micrometre-thick graphite films produced using high-temperature (3200 °C) of polyimide sheets^20^.

**Figure 6:**
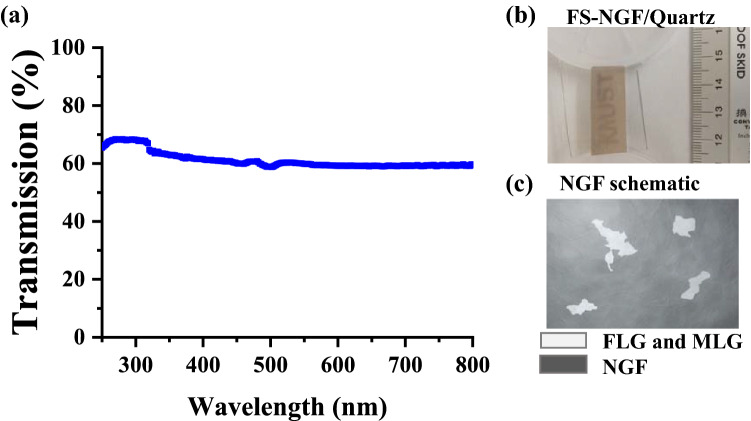
(**a**) UV–visible transmission measurements, (**b**) Typical NGF transfer on quartz with a typical sample. (**c**) Schematic of NGF (dark rectangle) with the well distributed FLG and MLG areas marked with gray random shapes (refer Fig. **1**) throughout the sample (roughly 0.1–3% areas in 100 µm^2^). The random shapes and their size in the schematic are merely illustrative and does not correspond to the actual areas.

We also performed UV–visible transmission measurements for the FS-NGFs transferred onto a quartz substrate (Fig. 6). The obtained spectrum showed an almost constant transmission of 62% in the range of 350–800 nm, indicating the NGF was semitransparent to visible light. In fact, the name ‘KAUST’ is visible as per the sample’s digital photo in Fig. 6b. Although the nanocrystalline structure of the NGF differs from that of SLG, one may roughly estimate the number of layers by using a rule of 2.3% transmittance loss for each additional layer^65^. Based on this relationship, the number of graphene layers for 38% transmission loss would be 21. The as-grown NGF consisted predominantly of 300 graphene layers, i.e., ~ 100 nm thickness (Fig. 1, SI5, and SI7). Therefore, we assume that the observable optical transparency corresponds to the FLG and MLG regions, as these are distributed all over the film (Figs. 1, 3, 5, and 6c). In addition to the above structural data, the conductivity and transparency properties confirm the high crystalline quality of the transferred NGF.

Semitransparent CVD grown NGFs were transferred onto bare Si surfaces and used in solar cells previously^15,16^. The power conversion efficiency (PCE) obtained was 1.5%. These NGFs serve multiple functions such as the active junction layer, a charge transport path, and as a transparent electrode^15,16^. However, the graphite films were non-uniform. Further optimization is required by carefully controlling the sheet resistance and the optical transmittance of the graphitic electrode, as both these properties play important roles in determining the PCE values of the solar cells^15,16^. Commonly, the graphene films have transparency of 97.7% to visible light, but their sheet resistance is 200–3,000 Ω/Sq^16^. The sheet resistance of the graphene film can be reduced by increasing the number of layers (perform multiple transfers of graphene layers) and by HNO_3_ doping (~ 30 Ω/sq)^66^. However, this process is time-consuming, and the different transferred layers are not always in good contact. Our front-side NGFs, with properties such as electrical conductivity of 2000 S/cm and sheet resistance of 50 Ω/sq film with 62% transparency, could be a viable alternative as a conducting channel or counter electrode in solar cells^15,16^.

#### BS-NGF: application in gas-sensing

Although the structure and surface chemistry of the BS-NGF was similar to that of FS-NGF, its roughness was different (“Growth of FS- and BS-NGF”). Previously, we used ultrathin films of graphite as gas sensors^22^. Therefore, we tested the possibility of using the BS-NGF for gas-sensing applications (Figure SI10). First, a mm^2^-sized section of BS-NGF was transferred onto an interdigitated electrode sensor chip (Figures SI10a–c). The chip fabrication details, with an active sensing area of 9 mm^2^, were reported previously^67^. In the SEM images (Figures SI10b and c), the underlying Au electrodes were clearly visible through the NGF. Likewise, it is seen that uniform chip coverage was achieved for all samples. Gas-sensing measurements (Figure SI10d) for various gases were recorded (Figure SI11), and the resulting responsivities are shown in Figure SI10g. It is evident that the device had excellent selectivity for NO_2_ (200 ppm) when compared to other interferent gases, including SO_2_ (200 ppm), H_2_ (2%), CH_4_ (200 ppm), CO_2_ (2%), H_2_S (200 ppm), and NH_3_(200 ppm). One plausible reason is the high electrophilic nature of the NO_2_ gas^22,68^. When adsorbed on the surface of graphene, it reduces the current electron uptake from the system. A comparison of response time data of the BS-NGF sensors with previously published sensors is presented in Table SI2. Further work on the reviving mechanism of the NGF sensors is ongoing considering use of UV light, O_3_ plasma, or heat (50–150 °C) treatments of the exposed samples. Ideally, an on-chip system^69^ will be realized afterward.

### Final discussion

Graphene growth occurs on both sides of a catalyst substrate in the CVD process^41^. However, BS graphene is usually discarded during the transfer process^41^. In this study, we have shown that high-quality NGF growth and polymer-free NGF transfer can be realized from both sides of a catalyst substrate. The BS-NGF was thinner (~ 80 nm) than the FS-NGF (~ 100 nm), a difference that was attributed to the BS-Ni not being directly exposed to the precursor gas flow. We also found that the roughness of the NiAR substrate affected the resulting NGF roughness. Those results suggest that the as-grown flat FS-NGFs could be used as source material for graphene (via an exfoliation method^70^) or as conducting channels in solar cell applications^15,16^. By contrast, the BS-NGFs would find use in gas-sensing (Figure SI9) and, possibly, energy strorage systems^71,72^, where its surface roughness would be beneficial.

Following the above, it is useful to contextualize the present work with previously reported thin films of graphite grown by CVD and using Ni foils. From Table 2, the higher pressure used by us allowed a much shorter reaction time (growth step), even at a relatively low temperature (where the interval is 850–1,300 °C). The area of growth we achieved is also larger than usual, pointing to possible scale-up. Other considerations can be made, some of which we included in the table.

**Table 2:** Comparison of the NGF growth process presented here with previously published articles of graphite films growth on Ni substrate using CVD technique.

Term used	Method	Substrate thickness (µm)	T (°C)	T_A_ (min); T_G_ (min)	P_c_ (mbar)	W (nm)	Growth area (cm^2^)	Transfer method; sample size (cm^2^)	R_s_ (Ω/sq); Tr (%)	Extra features/studies	Drawbacks/perspectives	1st Author, Journal, Year, Reference number
NGF	Cold wall CVD	Ni foil (25)	900	5; 5	500	100	55 (FS) 55 (BS)	Polymer- free; 6	50; 62	Wafer-scale double sided growth and transfer, free-standing on water, grain size 20–50 µm^2^, conductivity-2000 S/cm, gas- sensing application	Thickness uniformity need to be further improved at µm^2^ and nm^2^ scale by Ni surface engineering and other treatments	Current work, 2020
GF	Hot wall CVD	Ni foil (100)	1,150–1,000	15; 30	0.1	> 20	1	Polymer assisted; 1	NM	Wrinkle density reduced by Cl_2_ gas treatment to detach Ni at high temperature (1000ºC)	Long process (4 h), high temperature. non-uniform film, tears and holes present	Chatterjee, Chem. Mater. 2020 ^33^
GF	Hot wall CVD	Ni film (0.475) /Spinel	850–1,035	15; 90	66	22–2,254	0.01	Polymer assisted; NM	NM	Growth of single crystal graphite domains on Ni film on spinel substrate, GF grain size 10—100 µm	Limited area single crystal domains, long process time, surface defects present	Lu Cryst. Eng. Comm., 2020 ^64^
GF	Plasma CVD	Ni foil (30)	1,300	5; 60	0.2	350–380	1.6	Polymer assisted; 0.7	3.2; NM	Free-standing films, conductivity—10,000 S/cm, thermal conductivity measurements (1,570 W/mK)	Requires high sample temperature and plasma, limited area growth	Kato, Carbon, 2019 ^31^
NGF	Cold wall CVD	Ni foil (25)	1,035–860	40; 110	0.01	35–56	144	Polymer free; 4	65; 32	Large area growth using customized system, extreme ultraviolet mapping	Two stage growth, high temperature, long process time	Hu, Carbon, 2017, ^25^
GTF	Hot wall CVD	Ni (111) single crystal	900	30; 170	1.3	20–140	4 × 10^–6^	Polymer assisted; 4 × 10^–6^	NM	Thermal conductivity measurements (650–1,000 W/mK)	Expensive catalyst material, limited area growth, long process time	Zheng, Adv. Mater. Interface, 2016, ^5^
GF	Plasma CVD	Ni foil (500)	1,000	NM; 45	100	100–300	16	Polymer assisted; 4	NM	Free-standing film on 450 µm2 mesh, preferred orientation of folds showed, occurrence of blistered topology shown	Plasma and high growth temperature, high material cost and waste, longer growth time	Tyurnina, PSS, 2010, ^26^
TTG	Hot wall CVD	Ni foil or Film (NM)	1,000	30; 10–20	NM	10–100	0.1	Polymer assisted; 1	NM; 62–20	Semitransparent films, use of GF as conductive channels in solar cells	Nonuniform film, need to explore role of sheet-resistance and transmittance for solar cell applications	Li, Advanced materials, 2010 ^15^
GF	Cold wall CVD	Ni foil (50)	1,100	NM; 15	NM	300	2.5	Exfoliated SLG; 1.6 × 10^–6^	NM	Hall mobility of cleaved SLG, showed GF as source material for graphene research	High temperature growth, no details on cm^2^-scale thickness uniformity, limited area growth	Cai, Nano Res., 2009 ^70^
G	CVD	Ni film (0.5) /SiO_2_/Si	900–1,000	20; 5 -10	1,000	3	1–2	PMMA assisted; 0.4	770–1,000; 90	Ni grain after annealing 1–20 µm, conductivity 100–2000 S/cm	Nonuniform film, transferred film consists of broken areas	Reina, Nano letters, 2008 ^34^
GF	Hot wall CVD	Ni foil (100)	1,026–897	60; 10	100	0–60	1	NM	NM	Film growth using C_2_H_4_ precursor, TEM and SAED analysis	Nonuniform growth observed, TEM measurements done for dark regions, bright regions-No C	Johansson, TSF, 1994 ^32^

*G* graphene, *GF* Graphite film, *TTG* thin transparent graphite, *GTF* graphite thin film, *NM* not mentioned, *Tr*  Transmittance.

The sheet resistance is defined as R_s_ = 1/σW, where σ is the material conductivity and W- the film thickness.

## Conclusions

Double-sided, high-quality NGFs have been grown by catalytic CVD on Ni foils. By eliminating the conventional polymer support (as used in CVD graphene), we achieved a clean and defect-free wet-transfer of the NGFs (grown on the back- and front-sides of Ni foils) onto various technologically relevant substrates. Remarkably, the NGFs included FLG and MLG regions (nominally, 0.1 to 3% in 100 µm^2^) that were structurally well integrated in the overall thicker film. Plan-view TEM revealed that these regions were composed of stacks of two to three graphite/graphene grains (crystallites or layers, respectively), some having a rotational mismatch of 10°–20°. The FLG and MLG areas were responsible for the semitransparency of the FS-NGF to visible light. As for the back-side sheets, they could be transferred in parallel to the front-side ones and, as demonstrated, can have a functional purpose (e.g., for gas sensing). These studies bide well for the reduction of waste and costs in industrial-scale CVD processes.

Overall, the average thickness of the CVD NGFs lies in a region that falls between the (few- and multi-layer) graphene and the industrial (micrometer-thick) graphite sheets. Their array of interesting properties added to the straigtforward methods we developed to produce and transfer them, makes these films particularly well suited for applications that require the functional response of graphite without the costs of the currently used energy-intensive industrial production processes.

## Experimental methods

### NGF growth

A 25 µm Ni foil (99.5% purity, Goodfellow) was mounted in a commercial CVD reactor (4” BMPro Aixtron). The system was purged with Ar and evacuated to 10^–3^ mbar base pressure. The Ni foil was then heated to 900 °C, at a ramp rate of 75 °C/min in Ar/H_2_ (500/1,000 sccm, respectively) while maintaining a chamber pressure of 10 mbar. After pre-annealing the Ni foil for 5 min, the NGF was deposited by exposing the 900 °C foil to a CH_4_/H_2_ flow (100 sccm each) at 500 mbar for 5 min. The sample was then cooled at a rate of 40 °C/min to below 700 °C using an Ar flow (4,000 sccm). More details on the NGF growth process optimisation reported elsewhere^30^.

### Characterisation

The morphology of the sample surface was imaged via SEM, which was performed using a Zeiss Merlin microscope (1 kV, 50 pA). The sample surface roughness and NGF thickness were measured using AFM (Dimension Icon SPM, Bruker). TEM and SAED measurements were conducted with a FEI Titan 80–300 Cubed microscope equipped with a high-brilliance field emission gun (300 kV), a Wien-type FEI monochromator and a CEOS spherical aberration corrector (for the objective lens), which allowed a final spatial resolution of 0.09 nm. NGF samples were transferred to lacey-carbon coated Cu grids for plan-view TEM imaging and SAED pattern analysis. In this way, large portions of the sample flakes were suspended on the holes of the supporting film. The transferred NGF samples were analysed with XRD. The XRD patterns were obtained using a powder diffractometer (Brucker, D2 phaser with a Cu Kα source, 1.5418 Å and LYNXEYE detector) using a Cu radiation source with a beam spot diameter of 3 mm.

Several Raman spectroscopy point measurements were recorded with an integral confocal microscope (Alpha 300 RA, WITeC). A 532 nm laser with low excitation power (25%) was used to avoid heat-induced effects. The X-ray photoelectron spectroscopy (XPS) was carried out with a Kratos Axis Ultra spectrometer over the area of a 300 × 700 µm^2^ sample using monochromated Al Kα radiation (hν = 1,486.6 eV) at a power of 150 W. The survey and high-resolution spectra were acquired at pass energies of 160 eV and 20 eV, respectively. Transferred NGF samples on SiO_2_ were cut into pieces (each 3 × 10 mm^2^) using a PLS6MW Ytterbium Fiber laser (1.06 µm) with 30 W power. Cu wire (50 µm-thick) contacts were made, under an optical microscope, using Ag paste. Electrical transport and Hall effect experiments were carried out on these samples in a physical property measurement system (PPMS EverCool-II, Quantum Design, USA) at 300 K and by varying the magnetic field of ± 9 T. Transmission UV–Visible spectra were recorded in the range of 350 to 800 nm with a Lambda 950 UV–Visible spectrometer on the NGF transferred to a quartz substrate and reference quartz sample.

The chemi-resistance sensor (an inter-digitated electrode chip) was wire-bonded to a customised printed circuit board^73^, and the resistance was extracted in transient mode. The board, with the mounted device, was connected to contact leads and placed inside the gas-sensing chamber^74^. The resistance measurement was performed at 1 V, sweeping continuously from purge to gas exposure and then re-purging. Initially, the chamber cleaning was done by a purge with N_2_ gas at 200 sccm for 1 h, ensuring the depletion of all other analytes present in the chamber, including moisture. Thereafter, individual analytes were slowly released into the chamber at the same flow-rate of 200 sccm by closing the N_2_ cylinder.

## Supplementary information

Supplementary information

## References

[1] Inagaki, M. & Kang, F. *Materials Science and Engineering of Carbon: Fundamentals*. Second edition ed. 2014. 542.

[2] Pierson, H.O., *Handbook of carbon, graphite, diamond, and fullerenes: properties, processing, and applications*. 1st Edition ed. 1994, New Jersey.

[3] Cai W (2009). Large area few-layer graphene/graphite films as transparent thin conducting electrodes. Appl. Phys. Lett..

[4] Balandin AA (2011). Thermal properties of graphene and nanostructured carbon materials. Nat. Mater..

[5] Zheng QY, Braun PV, Cahill DG (2016). Thermal conductivity of graphite thin films grown by low temperature chemical vapor deposition on Ni (111). Adv. Mater. Interfaces.

[6] Hesjedal T (2011). Continuous roll-to-roll growth of graphene films by chemical vapor deposition. Appl. Phys. Lett..

[7] *Panasonic Graphite Sheets*. Available from: https://eu.mouser.com/datasheet/2/315/AYA0000CE2–64434.pdf.

[8] Saito, T., Y. Kihara, and J. Shirakashi, *Wearable strain sensors based on thin graphite films for human activity monitoring.* 6th International Conference on Materials and Applications for Sensors and Transducers, (Ic-Mast 2016), 2017. **939**.

[9] Joshi RK (2010). Graphene films and ribbons for sensing of O-2, and 100 ppm of CO and NO2 in practical conditions. J. Phys. Chem. C.

[10] Zeng YQ (2019). Thermally conductive reduced graphene oxide thin films for extreme temperature sensors. Adv. Funct. Mater..

[11] Shen B, Zhai WT, Zheng WG (2014). Ultrathin flexible graphene film: AN excellent thermal conducting material with efficient EMI shielding. Adv. Func. Mater..

[12] Kim TS (2017). Large area nanometer thickness graphite freestanding film without transfer process. Chem. Phys. Lett..

[13] Kim, M.J., et al., *Study of nanometer-thick graphite film for high-power EUVL pellicle.* Extreme Ultraviolet (Euv) Lithography Vii, 2016. **9776**.

[14] Kim SG (2015). Large-scale freestanding nanometer-thick graphite pellicles for mass production of nanodevices beyond 10 nm. Nanoscale.

[15] Li XM (2010). Graphene-on-silicon schottky junction solar cells. Adv. Mater..

[16] An XH, Liu FZ, Kar S (2013). Optimizing performance parameters of graphene-silicon and thin transparent graphite-silicon heterojunction solar cells. Carbon.

[17] Wang N (2018). Tailoring the thermal and mechanical properties of graphene film by structural engineering. Small.

[18] A. Tatami, et al. *Graphite thin films for accelertor applications*. in *Proceedings of the 14th Annual Meeting of Particle Accelerator Society of Japan*. August 1–3, 2017. Sapporo, Japan.

[19] Ragan S, Marsh H (1983). Review science and technology of graphite manufacture. J Mater. Sci..

[20] Murakami M, Tatami A, Tachibana M (2019). Fabrication of high quality and large area graphite thin films by pyrolysis and graphitization of polyimides. Carbon.

[21] Kim KS (2009). Large-scale pattern growth of graphene films for stretchable transparent electrodes. Nature.

[22] Deokar G (2020). Wafer-scale few-layer graphene growth on Cu/Ni films for gas sensing applications. Sens. Actuat. B Chem..

[23] Ferrari AC (2015). Science and technology roadmap for graphene, related two-dimensional crystals, and hybrid systems. Nanoscale.

[24] Kong W (2019). Path towards graphene commercialization from lab to market. Nat. Nanotechnol..

[25] Hu Q (2017). Large-scale nanometer-thickness graphite films synthesized on polycrystalline Ni foils by two-stage chemical vapor deposition process. Carbon.

[26] Tyurnina AV (2010). Topology peculiarities of graphite films of nanometer thickness. Phys. Status Solidi B Basic Solid State Phys..

[27] Obraztsov AN (2007). Chemical vapor deposition of thin graphite films of nanometer thickness. Carbon.

[28] *CVD graphene publications*. 2020; Available from: https://www.sciencedirect.com/search?qs=graphene%20CVD.

[29] Murata H (2019). High-electrical-conductivity multilayer graphene formed by layer exchange with controlled thickness and interlayer. Sci Rep.

[30] Deokar, G., A. Genovese, and Pedro M. F. J. Costa, *Fast, wafer-scale growth of a nanometer-thick graphite film on Ni foil and its structural analysis.* Nanotechnology, 2020. : PMID: 32679579, DOI: 10.1088/1361-6528/aba712 10.1088/1361-6528/aba712 32679579

[31] Kato R, Hasegawa M (2019). Fast synthesis of thin graphite film with high-performance thermal and electrical properties grown by plasma CVD using polycrystalline nickel foil at low temperature. Carbon.

[32] Johansson AS, Lu J, Carlsson JO (1994). Tem investigation of Cvd graphite on nickel. Thin Solid Films.

[33] Chatterjee S (2020). Synthesis of highly oriented graphite films with a low wrinkle density and near-millimeter-scale lateral grains. Chem. Mater..

[34] Reina A (2009). Large area, few-layer graphene films on arbitrary substrates by chemical vapor deposition. Nano Lett.

[35] Ma LP, Ren WC, Cheng HM (2019). Transfer methods of graphene from metal substrates: A review. Small Methods.

[36] Lee JH (2014). Wafer-scale growth of single-crystal monolayer graphene on reusable hydrogen-terminated germanium. Science.

[37] Chandrashekar BN (2015). Roll-to-roll green transfer of CVD graphene onto plastic for a transparent and flexible triboelectric nanogenerator. Adv. Mater..

[38] Deokar G (2015). Towards high quality CVD graphene growth and transfer. Carbon.

[39] Pham VP (2017). Direct growth of graphene on rigid and flexible substrates: Progress, applications, and challenges. Chem. Soc. Rev..

[40] Yang XJ, Yan MD (2020). Removing contaminants from transferred CVD graphene. Nano Res..

[41] Wei W (2015). Graphene FETs with aluminum bottom-gate electrodes and its natural oxide as dielectrics. IEEE Trans. Electron. Dev..

[42] Penezic A (2014). Carbohydrate-lectin interaction on graphene-coated surface plasmon resonance (SPR) interfaces. Plasmonics.

[43] Kim SG (2018). Formation process of graphite film on Ni substrate with improved thickness uniformity through precipitation control. Chem. Phys. Lett..

[44] Sun J (2010). Spatially-resolved structure and electronic properties of graphene on polycrystalline Ni. ACS Nano.

[45] Kozlova J (2015). Discontinuity and misorientation of graphene grown on nickel foil: Effect of the substrate crystallographic orientation. Carbon.

[46] Deokar G (2014). CVD graphene growth on Ni films and transfer, in 4th Graphene Conference, Graphene 2014.

[47] Hu QC (2019). A way to improve the uniformity of nanometer-thickness graphite film synthesized on polycrystalline Ni substrate: From large grain to small grain. Carbon.

[48] Wu XY (2016). Growth of continuous monolayer graphene with millimeter-sized domains using industrially safe conditions. Sci. Rep..

[49] Choi H (2012). Characterization of chemical vapor deposition-grown graphene films with various etchants. Carbon Lett..

[50] Kugeler K (1992). Aerosol formation by graphite corrosion in case of water and air ingress to the core of a high-temperature reactor. Nucl. Eng. Des..

[51] Hanaor D (2011). The effects of firing conditions on the properties of electrophoretically deposited titanium dioxide films on graphite substrates. J. Eur. Ceram. Soc..

[52] Boukhvalov DW (2014). Oxidation of a graphite surface: The role of water. J. Phys. Chem. C.

[53] Gotoh Y, Okada O (1984). C 1s binding-energy shift of pyrolytic-graphite bombarded by kev-deuterium ions. J. Nucl. Sci. Technol..

[54] Villa, N.D.A., *Metal-free Functionalized Carbons in Catalysis: Synthesis, Characterization*. 1st ed. RSC catalysis series. 2018, Cambridge: Royal Society of Chemistry.

[55] Retzko I, Unger WES (2003). Analysis of carbon materials by X-ray photoelectron spectroscopy and X-ray absorption spectroscopy. Adv. Eng. Mater..

[56] Dangelo M (2012). In-situ formation of SiC nanocrystals by high temperature annealing of SiO2/Si under CO: A photoemission study. Surf. Sci..

[57] Shin YJ (2010). Surface-energy engineering of graphene. Langmuir.

[58] Rubes M (2009). Structure and stability of the water-graphite complexes. J. Phys. Chem. C.

[59] Rafiee J (2010). Superhydrophobic to superhydrophilic wetting control in graphene films. Adv. Mater..

[60] Pagics, A., et al., *THz Near-Field Nanoscopy of Graphene Layers.* 2015 40th International Conference on Infrared, Millimeter and Terahertz Waves (Irmmw-Thz), 2015.

[61] Aslin J (2019). Ripplocations provide a new mechanism for the deformation of phyllosilicates in the lithosphere. Nat. Commun..

[62] Gruber J (2016). Evidence for bulk ripplocations in layered solids. Sci. Rep..

[63] Ramnani P (2017). Raman spectra of twisted CVD bilayer graphene. Carbon.

[64] Lu ZH (2020). Large scale epitaxial graphite grown on twin free nickel(111)/spinel substrate. CrystEngComm.

[65] Nair RR (2008). Fine structure constant defines visual transparency of graphene. Science.

[66] Bae S (2010). Roll-to-roll production of 30-inch graphene films for transparent electrodes. Nat. Nanotechnol..

[67] Yassine O (2016). H2 S sensors: Fumarate-based fcu-MOF Thin film grown on a capacitive interdigitated electrode. Angew Chem. Int. Ed Engl.

[68] Vijjapu MT (2020). Fully integrated indium gallium zinc oxide NO2 gas detector. ACS Sens..

[69] Yuvaraja S (2020). Fully integrated organic field-effect transistor platform to detect and to quantify NO2 gas. Phys. Status Solidi-Rapid Res. Lett..

[70] Cai WW (2009). Synthesis of isotopically-labeled graphite films by cold-wall chemical vapor deposition and electronic properties of graphene obtained from such films. Nano Res..

[71] Ru Y (2019). Different positive electrode materials in organic and aqueous systems for aluminium ion batteries. J. Mater. Chem. A.

[72] Li Z (2018). A novel graphite-graphite dual ion battery using an AlCl3–[EMIm]Cl liquid electrolyte. Small.

[73] Yuvaraja S (2020). Realization of an ultrasensitive and highly selective OFET NO2 sensor: The synergistic combination of PDVT-10 polymer and porphyrin-MOF. ACS Appl. Mater. Interfaces..

[74] Surya S (2019). Silver nanoparticles anchored UiO-66 (Zr) metal-organic framework (MOF) based capacitive H2S gas sensor. CrystEngComm.

