# Publisher Correction: Semi-transparent graphite films growth on Ni and their double-sided polymer-free transfer

**DOI:** 10.1038/s41598-020-75524-5

**Published:** 2020-10-29

**Authors:** Geetanjali Deokar, Alessandro Genovese, Sandeep G. Surya, Chen Long, Khaled N. Salama, Pedro M. F. J. Costa

**Affiliations:** 1 Physical Science and Engineering Division, King Abdullah University of Science and Technology (KAUST), Thuwal, 23955-6900 Saudi Arabia; 2 Core Labs, King Abdullah University of Science and Technology, Thuwal, 23955-6900 Saudi Arabia; 3 Sensors Lab, Advanced Membranes and Porous Materials Center, Computer, Electrical and Mathematical Science and Engineering Division, King Abdullah University of Science and Technology, Thuwal, 23955-6900 Saudi Arabia

Correction to: *Scientific Reports* 10.1038/s41598-020-71435-7, published online 07 September 2020

This Article contains an error in Figure 2, where a corruption has affected panels (d) and (e). The correct Figure 2d, e appears below as Figure 1.

**Figure 1:**
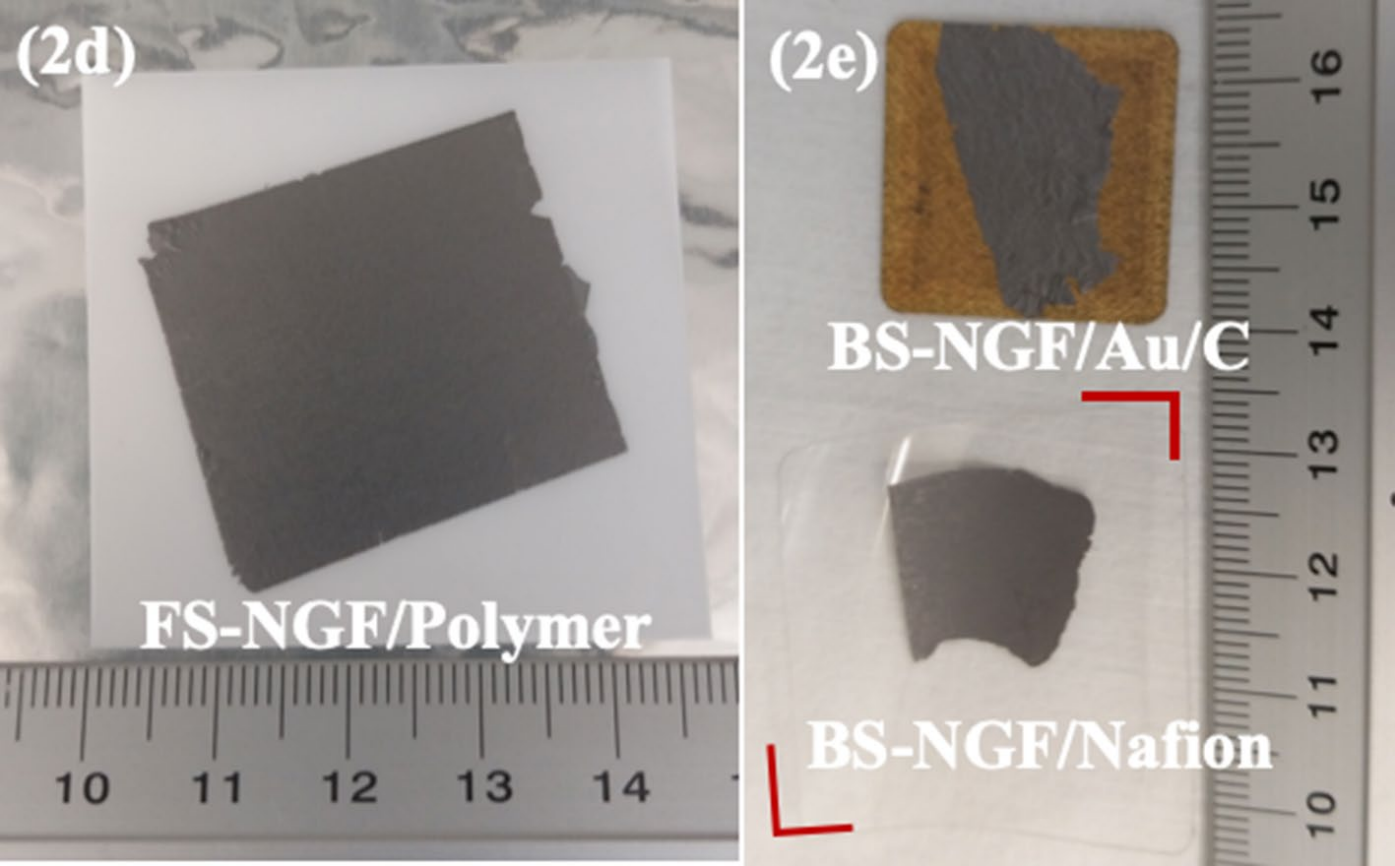
A correct version of the original Figure 2d, e.

