# Supplementary material for: Semi-transparent graphite films growth on Ni and their double-sided polymer-free transfer
Source: Sci Rep. 2020 Sep 7;10:14703. doi: 10.1038/s41598-020-71435-7 (PMC7477098; doi:10.1038/s41598-020-71435-7)
Supplement: Supplementary file 1 — Supplementary information [file 41598_2020_71435_MOESM1_ESM.docx]

**Semi-transparent graphite films growth on Ni and their double-sided polymer-free transfer**

Geetanjali Deokar^1*^, Alessandro Genovese^2^, Sandeep G. Surya ^3^_,_ Chen Long^2^_,_ Khaled N. Salama^3^, Pedro M. F. J. Costa^1^

^1^ King Abdullah University of Science and Technology (KAUST), Physical Science and Engineering Division, Thuwal, 23955‐6900, Saudi Arabia

^2^ King Abdullah University of Science and Technology, Core Labs, Thuwal, 23955-6900, Saudi Arabia

^3.^ King Abdullah University of Science and Technology, Sensors lab, Advanced Membranes and Porous Materials Center, Computer, Electrical and Mathematical Science and Engineering Division, Thuwal, 23955-6900, Saudi Arabia

Corresponding author^*^: [geetanjali.deokar@kaust.edu.sa](mailto:geetanjali.deokar@kaust.edu.sa)

**Suplementory Information:**

**Table SI1: Raman spectra analysis for FS- and BS-NGF/Ni**

**
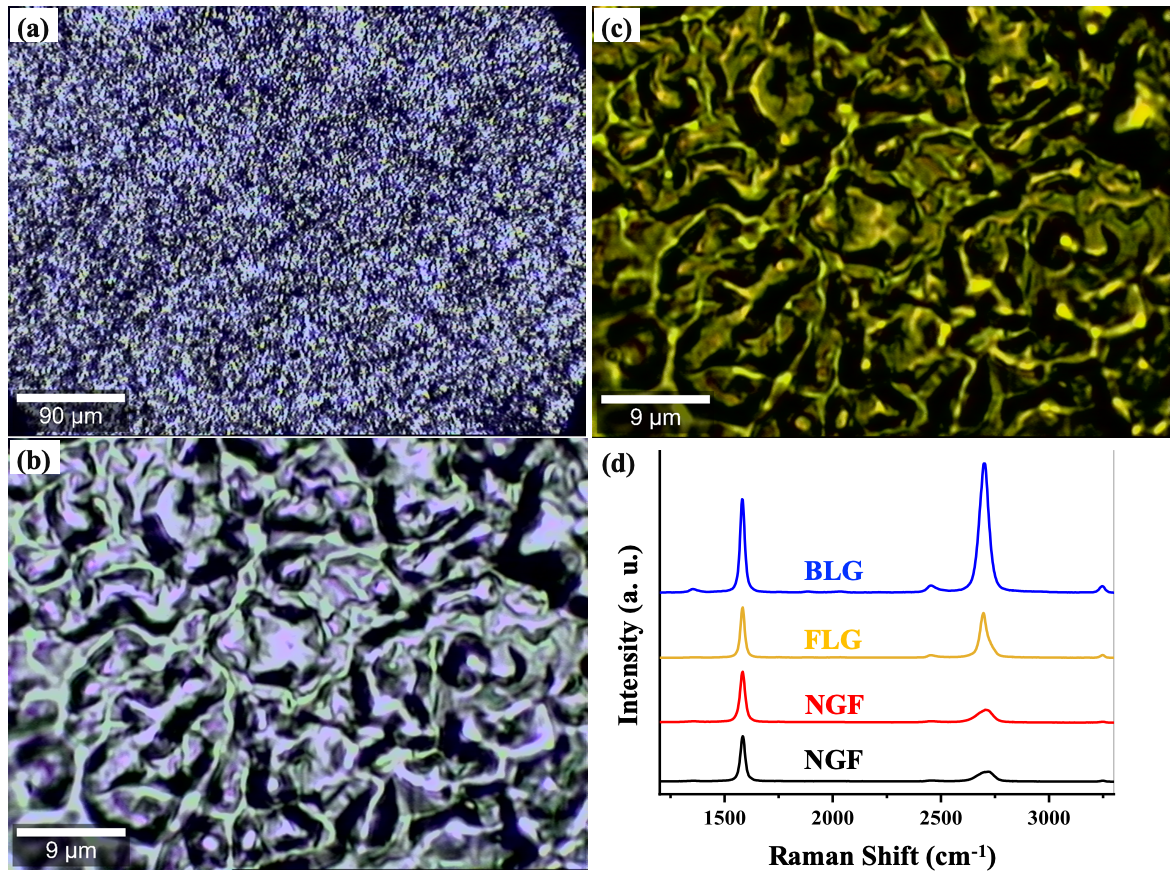
**

**Figure SI1.** BS-NiAG/Ni: (a-b) Optical microscopy images recorded with use of white light, (c) Optical image using green light filter at same position as that of panel-b. Typical Raman spectra recorded on image in panel-b. Unlike FS-NGF/Ni, it was difficult to corelate the bright and dark regions with Raman spectrum. Raman spectra were collected by simply changing positions by X-Y position scanner, and typical point spectra were collected.

**
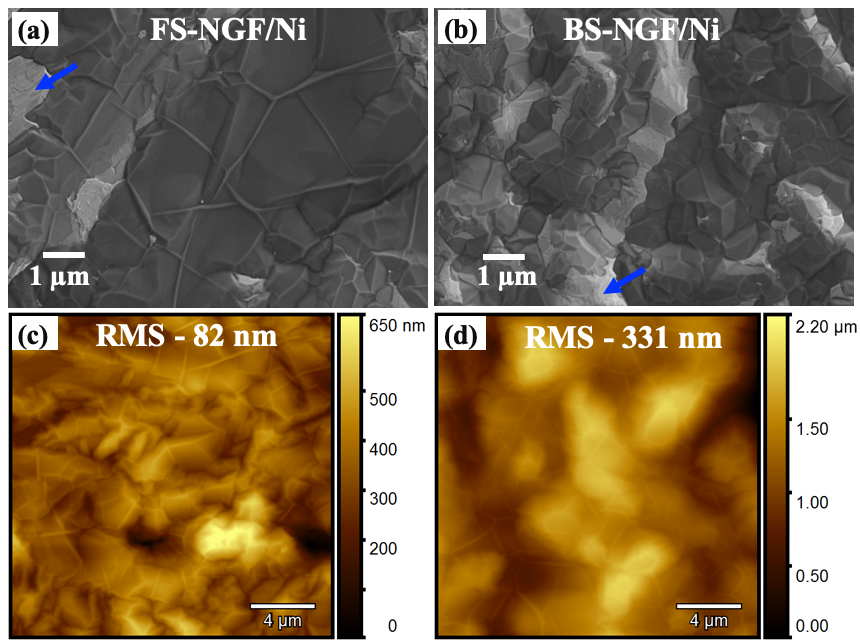
**

**Figure SI2.** Comparison of FS- and BS-NiAG/Ni samples: (a) and (b) SEM images on FS-NGF/Ni and BS-NGF/Ni, respectively. (c) and (d) AFM images (20 x 20 µm^2^) of FS-NGF/Ni and BS-NGF/Ni. Regions with steps on Ni surface visible in the FLG areas marked by blue arrow in panel-a and panel-b.

**
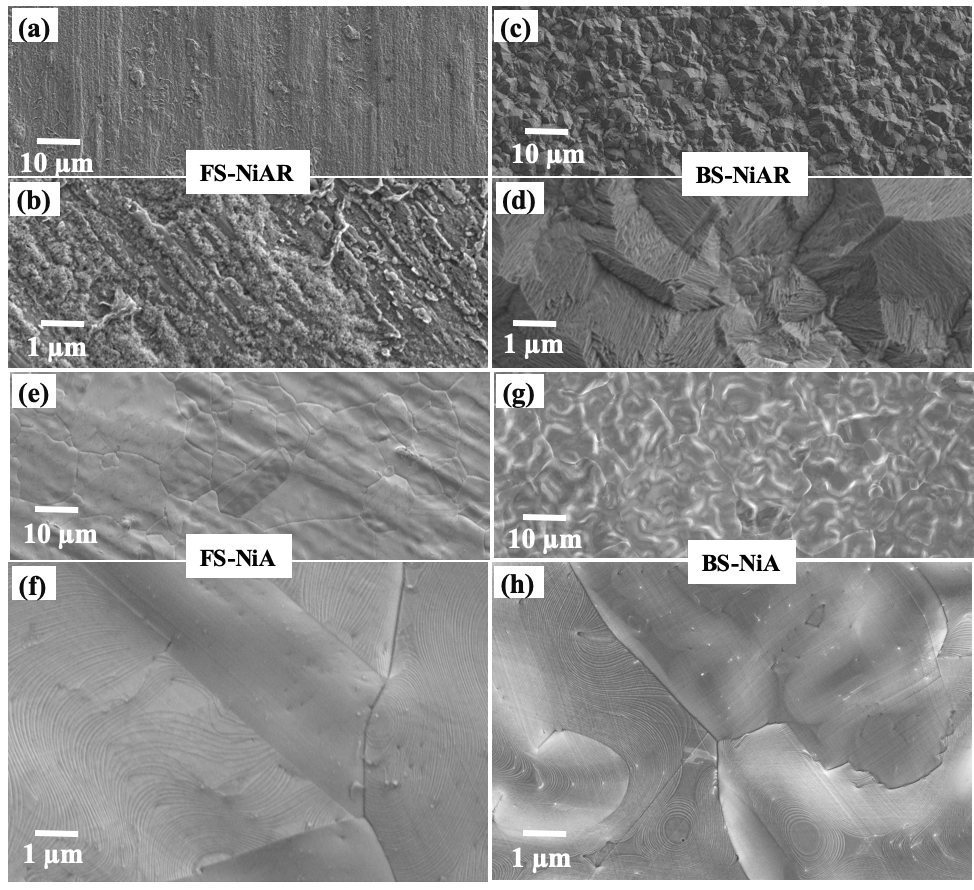
**

**Figure SI3.** Low- and high-magnification SEM images of NiAR and NiA foils. (a, b) FS-NiAR foil. (c, d) BS-NiAR foil. (e, f) FS-NiA foil. (g, h) BS-NiA foil.

**
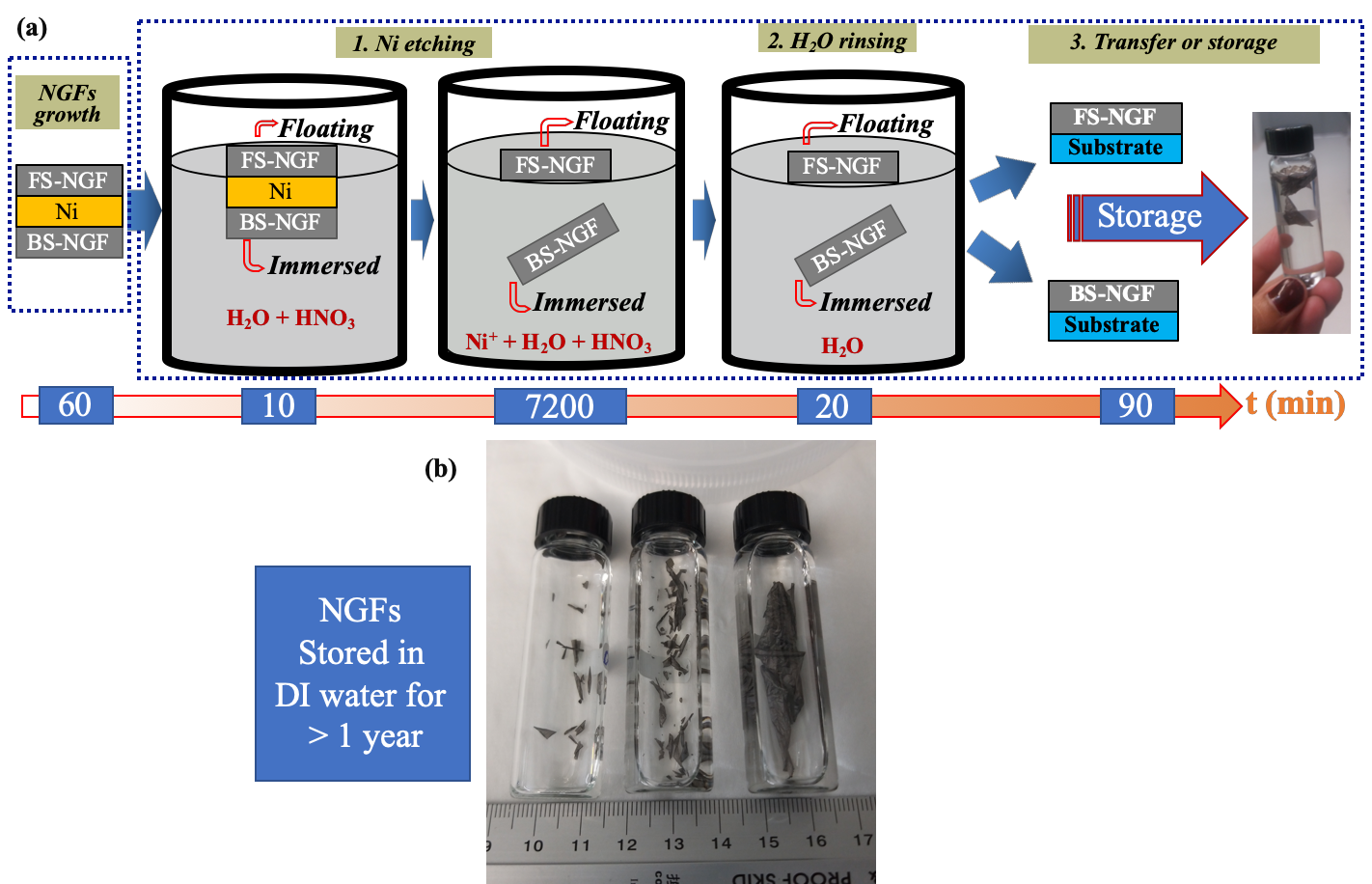
**

**Figure SI4.** (a) NGF growth and polymer-free wet-chemical transfer process time-line, (b) Samples storage at room temperature in DI water and sealed bottles (>1 year) big and small flakes collections.

**
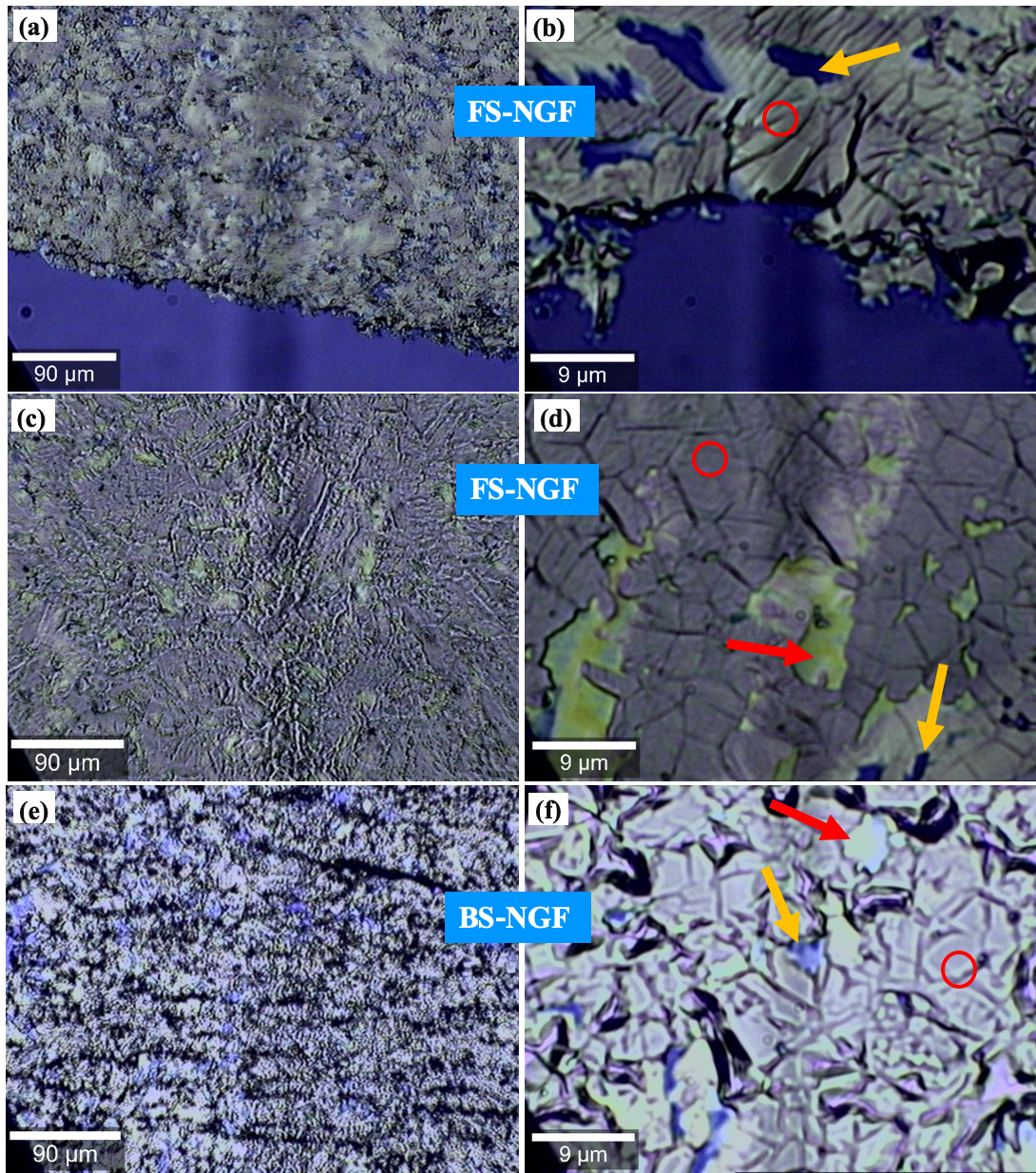
**

**Figure SI5.** Optical imaging (OM) of FS- and BS-NGF on SiO_2_(300 nm)/Si samples (recorded with use of white light): (a-b), (c-d) OM images on FS-NGF at the edge and in the center of the sample. (e-f) OM images on BS-NGF in the center of the sample. Dominantly present NGF areas marked by red circle in panel-b, panel-d and panel-f. FLG areas can be identified by blue regions indicated by arrows. MLG areas by orange arrows.

**
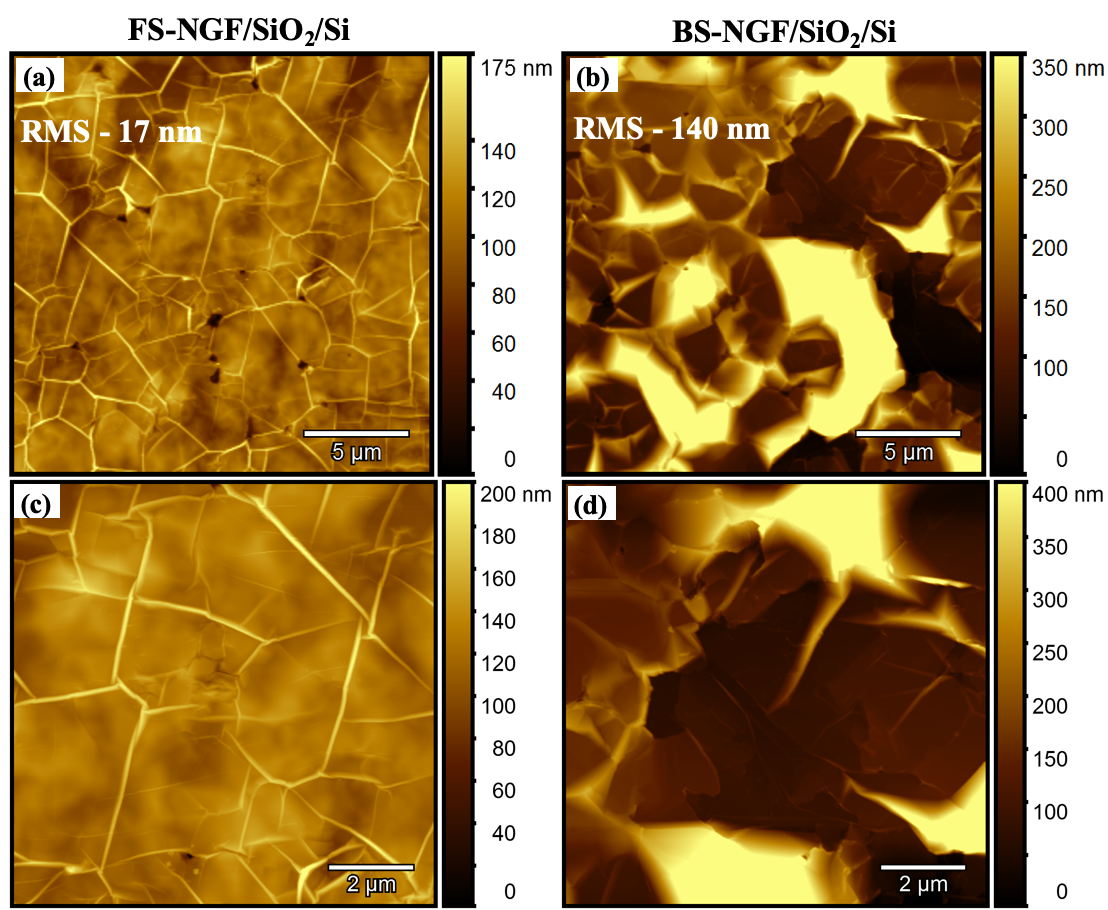
**

**Figure SI6.** AFM images of transferred NiAG sample on SiO_2_/Si large (20 x 20 µm^2^) and high-magnification (10 x 10 µm^2^) scans: **(**a, b) FS-NGF, **(**c, d) BS-NGF.


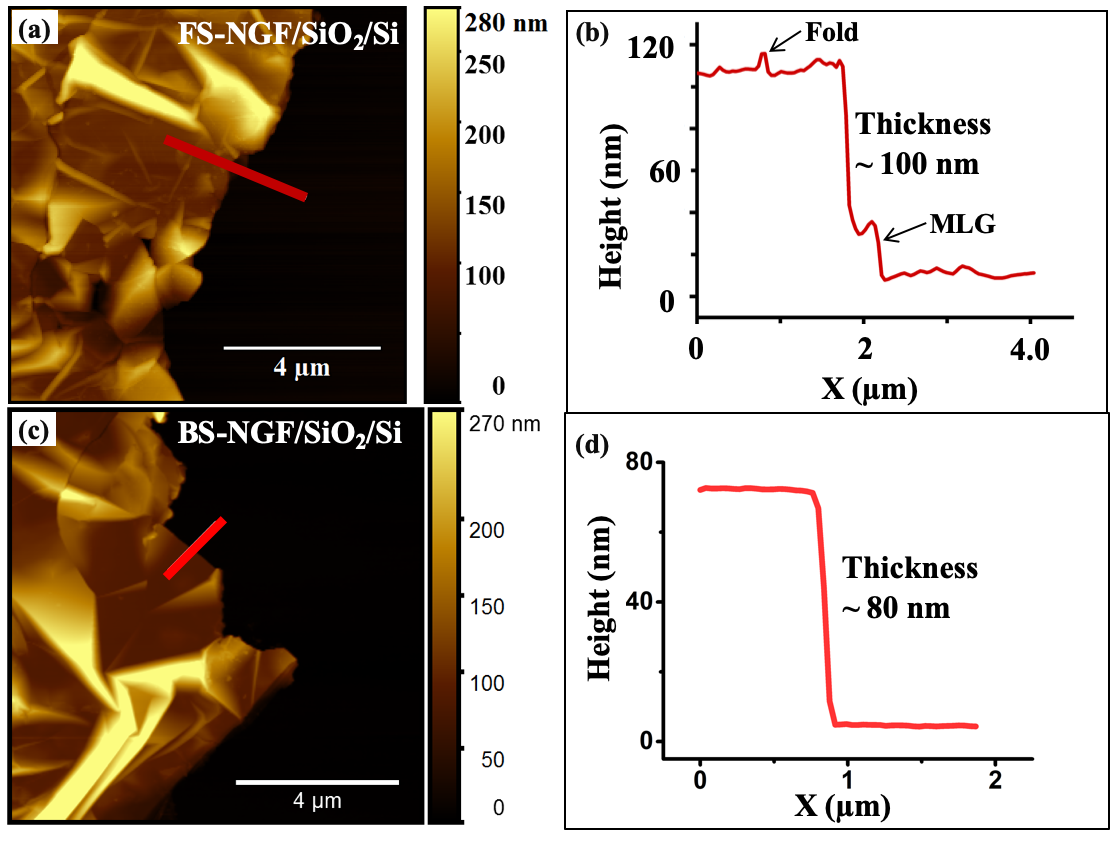


**Figure SI7.** (a) AFM images FS-NGF/SiO_2_/Si at the edge, (b) Hight profile along the red line in panel-a. (c) AFM image BS-NGF/SiO_2_/Si at the edge. (d) Hight profile along the red line in panel-c.


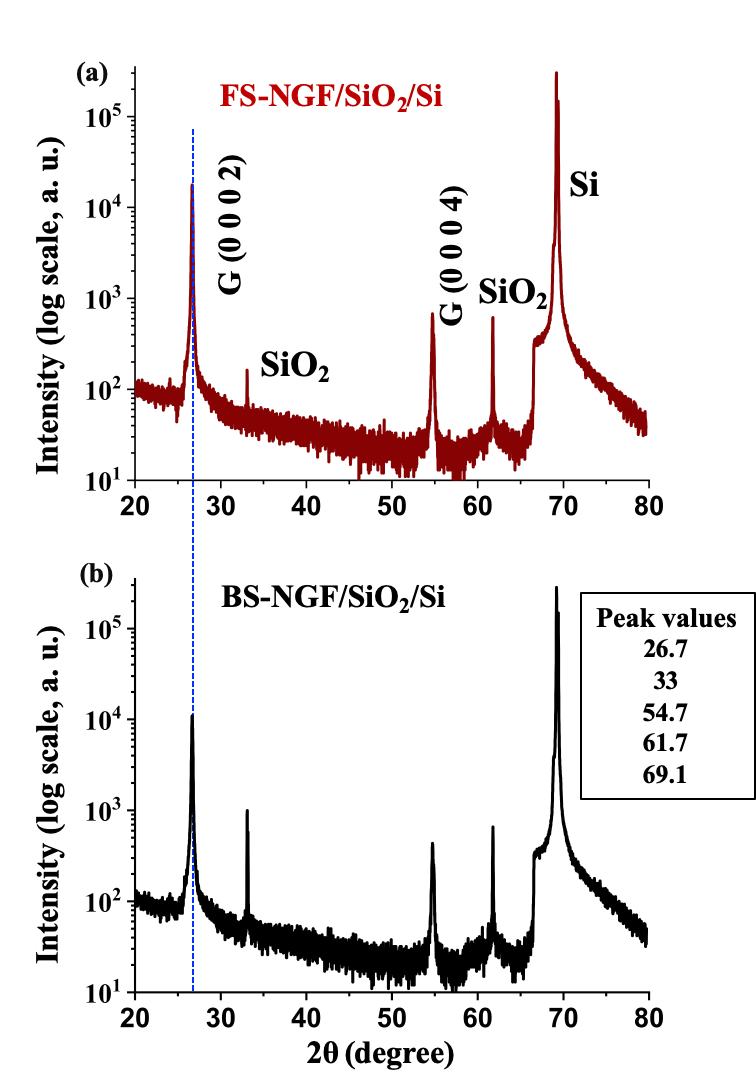


**Figure SI8.** Typical XRD spectra of NGF/SiO_2_/Si substrate: **(**a) FS-NGF/SiO_2_, (b) BS-NGF/SiO_2_. Full width half maximum for G(0 0 0 2) peak is 0.4º for both samples.


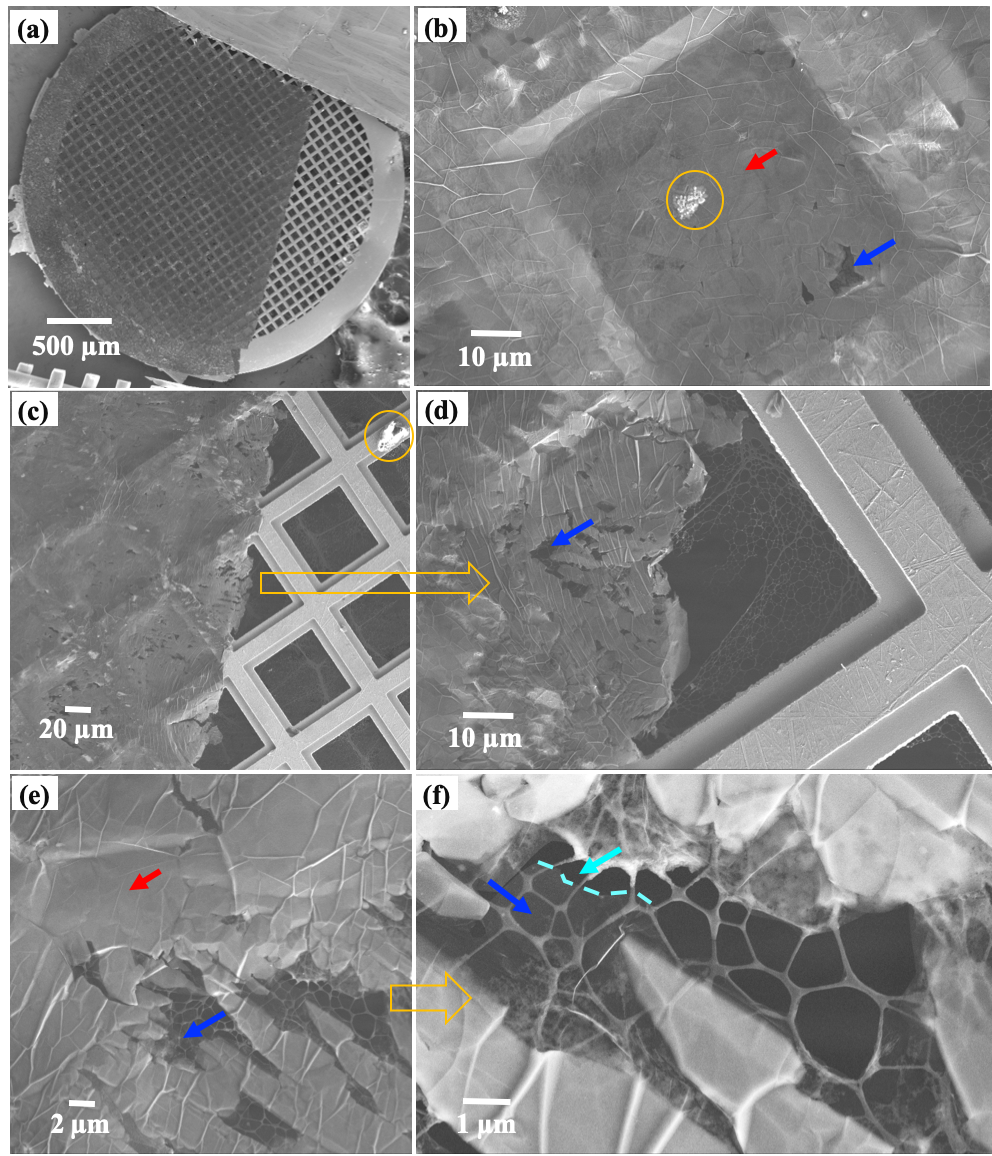


**Figure SI9.** SEM images (recorded after TEM measurements as presented in **Figure 5**) of polymer-free NGF transferred on Cu grid with lacey carbon. (a) Low-magnification SEM image of the sample. (b) NGF suspended on lacey carbon, (c) SEM image at the edge of NGF on Cu grid, orange circle marks presence of some dust particles on Cu grid. (d) High-magnification SEM image at the NGF edge showing NGF and lacey carbon, (e) SEM image showing NGF and FLG areas with corresponding high-magnification image in panel-f. Red and blue arrows indicate NGF and FLG areas, respectively. Cyan arrow and dotted cyan line indicate a broken FLG film and it’s boundary, respectively.


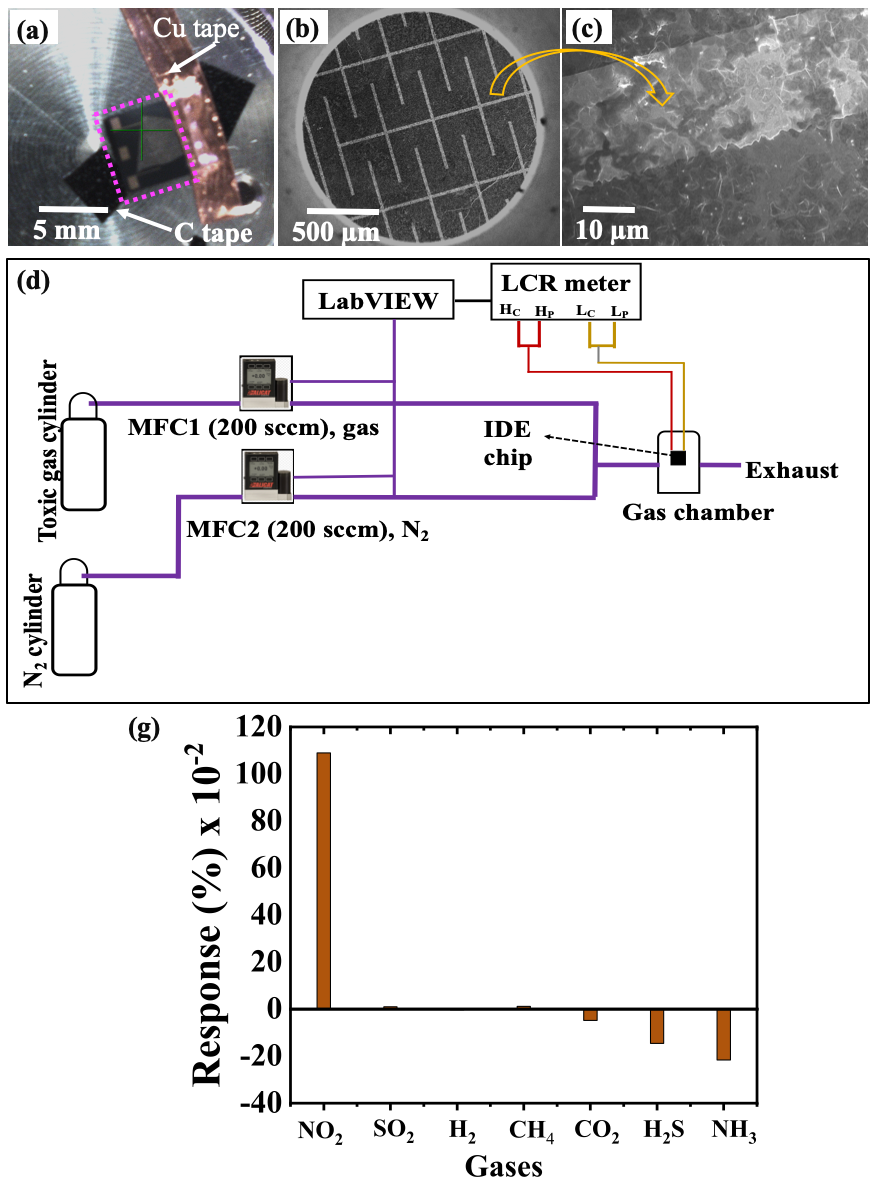


**Figure SI10.** (a) Typical interdigitated electrodes (IDE) sensor device (marked with dotted magenta rectangle) with BS-NGF, gray film close to the green + sign, sample photo taken in navigation mode during SEM imaging. (b) Low-magnification SEM image on the sample in panel-a (NGF on IDE). (c) High-magnification SEM image close to one of the IDE. (d) Schematic of gas detection setup with the chamber and measurement unit. (e) Gas detection response (%) measured for various reducing and oxidizing gases.

**
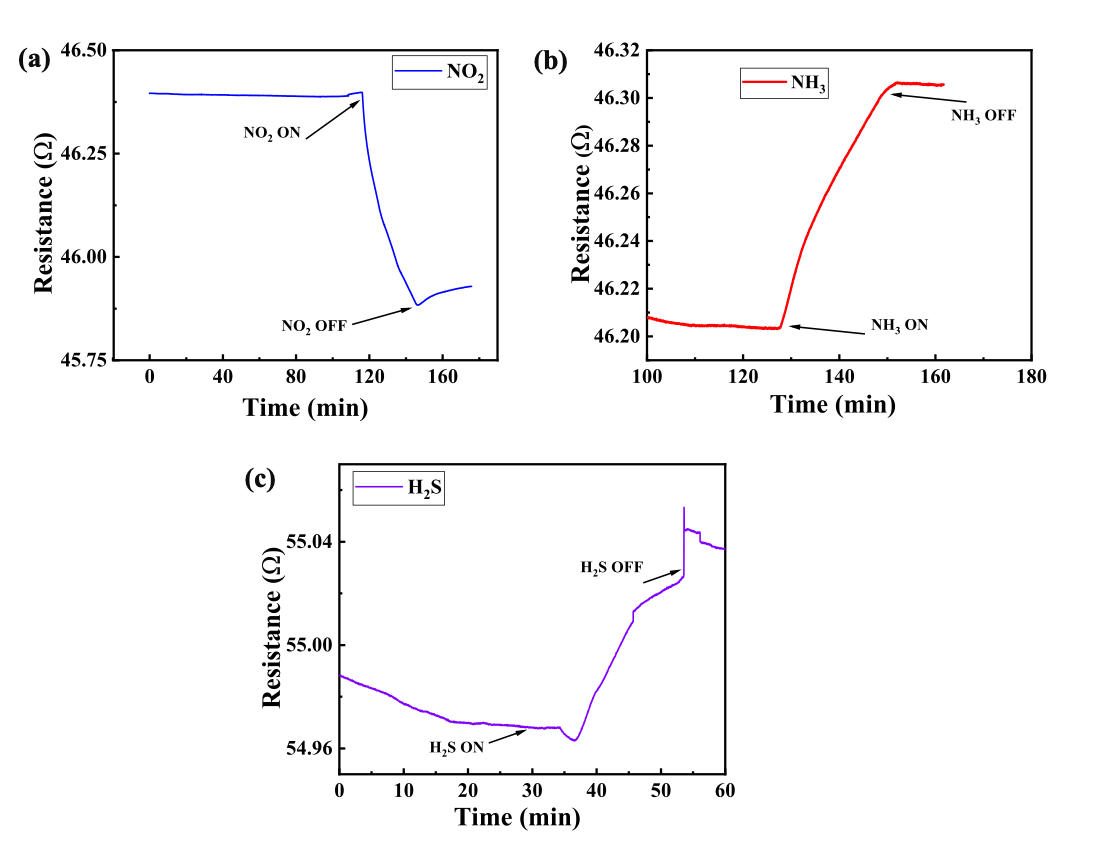
**

**Figure SI11**. Response time data of the BS-NGF sensors for (a) NO_2_ gas (b) NH_3_, and (c) H_2_S

| **Active material*** | **LOD**  **(ppm)** | **Sensing condition** | **Recovery condition** | **Interferent gases used** | **Refs** |
| --- | --- | --- | --- | --- | --- |
| TiO_2_ (CR) | 100 | RT | RT(Air) | CO, CO_2_ | 1 |
| IGZO (TFT ) | 0.5 | RT and 100 ^o^C | 100 ^o^C | No selectivity | 2 |
| IGZO (TFT) | 2 | RT(UV ) | RT(UV) | No selectivity | 3 |
| MOS_2_  (Pt decorated) | 0.5 | RT | RT | No selectivity | 4 |
| PCDTBT (OFET) | 1 | RT | RT | H_2_S, NH_3_, NO | 5 |
| NGF (CR)* | 50 | RT | Non-recoverable | CO_2_, SO_2_, CH_4_, H_2_, H_2_S and NH_3_ | This work |

**Table SI1.** A comparison of response of the BS-NGF sensors with previously published sensors (CR – chemi-resistive; TFT-thin-film transistor; OFET – organic transistor)

**References:**

1. Xie, T., Sullivan, N., Steffens, K., Wen, B., Liu, G., Debnath, R., ... & Motayed, A. (2015). UV-assisted room-temperature chemiresistive NO2 sensor based on TiO2 thin film. *Journal of alloys and compounds*, *653*, 255-259.

2. Kim, K. S.; Ahn, C. H.; Jung, S. H.; Cho, S. W.; Cho, H. K. Toward Adequate Operation of Amorphous Oxide Thin-Film Transistors for Low-Concentration Gas Detection. *ACS Appl Mater Interfaces* **2018,** *10* (12), 10185-10193, DOI: 10.1021/acsami.7b18657.

3. Knobelspies, S.; Bierer, B.; Daus, A.; Takabayashi, A.; Salvatore, G. A.; Cantarella, G.; Ortiz Perez, A.; Wollenstein, J.; Palzer, S.; Troster, G. Photo-Induced Room-Temperature Gas Sensing with a-IGZO Based Thin-Film Transistors Fabricated on Flexible Plastic Foil. *Sensors (Basel)* **2018,** *18* (2), DOI: 10.3390/s18020358.

4. Hong, H. S.; Phuong, N. H.; Huong, N. T.; Nam, N. H.; Hue, N. T. Highly sensitive and low detection limit of resistive NO2 gas sensor based on a MoS2/graphene two-dimensional heterostructures. Applied Surface Science 2019, 492, 449-454,

5. Kumar, A.; Jha, P.; Singh, A.; Chauhan, A. K.; Gupta, S. K.; Aswal, D. K.; Muthe, K. P.; Gadkari, S. C. Modeling of gate bias controlled NO 2 response of the PCDTBT based organic field effect transistor. Chemical Physics Letters 2018, 698, 7-10
